# Chromatographic Fingerprinting Enables Effective Discrimination and Identitation of High-Quality Italian Extra-Virgin Olive Oils

**DOI:** 10.1021/acs.jafc.1c02981

**Published:** 2021-07-28

**Authors:** Federico Stilo, Ana M. Jiménez-Carvelo, Erica Liberto, Carlo Bicchi, Stephen E. Reichenbach, Luis Cuadros-Rodríguez, Chiara Cordero

**Affiliations:** † Dipartimento di Scienza e Tecnologia del Farmaco, Università degli Studi di Torino, Via Pietro Giuria 9, Torino I-10125, Italy; ‡ Department of Analytical Chemistry, Faculty of Science, University of Granada, Av. Fuentenueva S/N, Granada E-18071, Spain; § University of Nebraska, Lincoln, Nebraska 68588, United States; ∥ GC Image LLC, Lincoln, Nebraska 68508, United States

**Keywords:** comprehensive two-dimensional gas chromatography, extra-virgin olive oil, combined untargeted and targeted (UT) fingerprinting, identitation and authentication, geographical origin

## Abstract

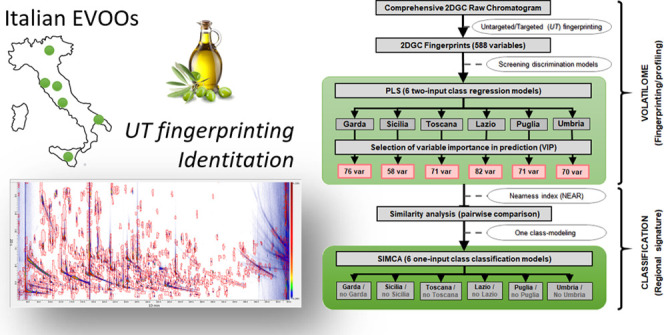

The challenging process of high-quality food authentication takes advantage of highly informative chromatographic fingerprinting and its identitation potential. In this study, the unique chemical traits of the complex volatile fraction of extra-virgin olive oils from Italian production are captured by comprehensive two-dimensional gas chromatography coupled to time-of-flight mass spectrometry and explored by pattern recognition algorithms. The consistent realignment of untargeted and targeted features of over 73 samples, including oils obtained by different olive cultivars (*n* = 24), harvest years (*n* = 3), and processing technologies, provides a solid foundation for sample identification and discrimination based on production region (*n* = 6). Through a dedicated multivariate statistics workflow, identitation is achieved by two-level partial least-square (PLS) regression, which highlights region diagnostic patterns accounting between 58 and 82 of untargeted and targeted compounds, while sample classification is performed by sequential application of soft independent modeling for class analogy (SIMCA) models, one for each production region. Samples are correctly classified in five of the six single-class models, and quality parameters [i.e., sensitivity, specificity, precision, efficiency, and area under the receiver operating characteristic curve (AUC)] are equal to 1.00.

## Introduction

Olive oil (OO) is one of the pillars of the Mediterranean diet and represents the main source of fats in the countries of the Mediterranean basin.^1^ In particular, extra-virgin olive oil (EVOO) is recognized as the most valuable product among the edible oils;^2^ it is extracted from fresh olive fruits (*Olea europeae* L.) by mechanical or physical technologies that preserve the composition of the lipid fraction while limiting autoxidation reactions and alterations of its native quality.^3^

The reason for the increasing demand for olive oil of high quality, i.e., EVOO (Commission of the European Communities, 1991; “EU Food Qual. Labels”, 2021; IOC, 2015), is not only because of its nutritional and health values, due to the presence of antioxidants (i.e., tocopherols and phenolic compounds) and high oleic acid content, but also because of its peculiar sensory characteristics^2^ strongly related to olive cultivar, pedoclimatic conditions of the harvest region, olive ripeness, and extraction technology (EU Food Qual. Labels, 2021).

In this context, any analytical methodology capable of delineating chemical patterns informative of the different functional variables influencing the composition of EVOO is useful and has the potential to support the valorization of high-quality products, facilitate sensory quality evaluation/screenings on the basis of commercial classification, as well as to counteract fraudulent practices.^4−7^ In this latter context, the accurate fingerprinting of the unsaponifiable fraction and of minor components by comprehensive two-dimensional (2D) gas chromatography coupled to mass spectrometry (GC × GC–MS) and/or to parallel flame ionization detection (MS/FID) was successful in identifying admixtures of OO with other fats and/or establish the product freshness/shelf life.^8^

Active research in the development of GC fingerprinting methodologies includes also investigations on EVOO volatiles. Monodimensional (1D)-GC fingerprinting accompanied by accurate profiling was recently applied to validate the role of sesquiterpene hydrocarbons as geographical origin markers^7^ in EVOOs from different cultivars and production areas. In their study, Quintanilla-Casas et al.^7^ confirmed the superior discrimination power of the total volatile fingerprint (100% correct classification) obtained by GC–MS raw data processing followed by suitable supervised chemometrics, compared to the targeted profiling of selected sesquiterpenoids, whose correctness ranged between 46 and 100% of the correct classification, as a function of the production country.

Moreover, using volatile fingerprinting based on 1D-GC, it was also possible to support the commercial classification of OO based on sensory panel evaluation.^9^ EVOOs are in fact characterized by peculiar yet essential aroma qualities such as green, grassy, and fruity notes, whose perception is at the basis of commercial classification based on EU regulations. Sensory quality in fact, together with compositional/chemical standards to be complied,^5^ and absence of off-flavors guide the OO classification in EVOO (median of the defects = 0.0 and median of the positive attribute > 0.0), virgin olive oil (VOO—median of the defects between 0.0 and 3.5 and median of the positive attribute > 0.0), and lampante olive oil (LOO—median of the defects > 6.0) in the presence of sensory defects (rancid, fusty/muddy, musty, and winey/vinegary), even at lower levels (e.g., median of the defects ≠ 0).^5^

In this study, we take a step forward in the direction of validating a powerful and highly flexible chromatographic fingerprinting workflow with superior identitation (i.e., defining the identity of a particular food based on the characteristic features that make it singular or unique^10^) and classification effectiveness compared to existing tools based on 1D-GC data. The improved separation capacity of GC × GC, the analyte retention logic over the separation space,^11^ and the comprehensive capture of a component’s features generated by time-of-flight mass spectrometry (TOF MS) detection make the resulting 2D fingerprints the sample’s unique traits for effective and reliable authentication. Moreover, the specificity of the third information dimension of the system (i.e., EI-MS fragmentation patterns) gives access to a higher informative level as any confirmatory analytical technique.

Compared to existing studies adopting GC × GC as the profiling and/or fingerprinting technique,^12−14^ the combined information derived from untargeted and targeted (UT) features is here explored in the challenging scenario of Italian high-quality EVOO production connoted by an impressive heritage of olive genetic varieties, with about 540 different registered cultivars^15^ and 46 protected designation of origin (PDO) products from different geographical locations (i.e., regions) over the entire territory.

The challenge posed by the complexity and high chemical dimensionality of EVOO volatiles is tackled by a dedicated workflow, named combined untargeted and targeted fingerprinting (UT fingerprinting),^16^ where the information from known and unknown components patterns are accurately tracked across many samples and their identitation, discrimination, and classification power is examined in great detail with a focus on regional characters. Furthermore, the synergy between profiling and fingerprinting is also examined by observing the distribution of key-aroma compounds and potent odorants strongly correlated to positive and/or negative odor qualities.^17−19^

## Materials and Methods

### Chemicals

Pure reference standards of α- and β-thujone and methyl-2-octynoate used as internal standards (ISs), *n*-alkanes (from *n*-C7 to *n*-C25) used for linear retention index (*I*^T^) calibration, and pure reference compounds for identity confirmation were supplied by Merck (Milan, Italy). Cyclohexane (HPLC grade) for *n*-alkane dilution and pure dibutyl phthalate used to prepare IS working solutions were also from Merck (Milan, Italy).

### Extra-Virgin Olive Oil Samples

Extra-virgin olive oils (EVOOs) were supplied within the VIOLIN project^20^ selection. They were obtained from olives of different cultivars harvested between 2016 and 2018 over the Italian territory; all samples were certified as EVOOs by accredited laboratories (ISO 17025:2018) and by the official sensory panel test. Details on the sample set under study, counting 73 samples, are provided in the Supporting Information Table S1 together with harvest/production regions (i.e., Umbria *n* = 7, Garda lake *n* = 10, Lazio *n* = 11, Puglia *n* = 12, Sicilia *n* = 13, and Toscana *n* = 20). Supporting Information Figure S1 shows geographical locations of selected EVOO production sites.

### Headspace (HS) Solid-Phase Microextraction (SPME) Devices and Sampling Conditions

Volatiles were sampled by headspace (HS) solid-phase microextraction (SPME). A divinylbenzene/carboxen/polydimethylsiloxane (DVB/CAR/PDMS) *d*_f_ 50/30 μm 2 cm length fiber (Supelco, Bellefonte, PA) was chosen based on its sampling effectiveness on EVOO volatiles and previous research.^17,21−23^ SPME fibers were conditioned before use as recommended by the manufacturer.

The ISs were preloaded onto the SPME device by sampling 5.0 μL of α/β-thujone and methyl-2-octynoate IS solution (100 mg L^–1^) placed in a 20 mL headspace vial. IS preloading was performed by exposing the SPME device to the HS kept at 40 °C for 5 min.

Sampling was carried out on 0.100 ± 0.005 g of oil samples, precisely weighed in 20 mL headspace vials, at 40 °C for 60 min under constant stirring. The amount of sample was chosen matching for HS linearity conditions for most of the characteristic analytes of the EVOO volatile fraction.^21,24,25^ After extraction, the SPME device was automatically transferred to the split/splitless injection port of the GC × GC system kept at 250 °C, and thermal desorption was for 5 min.

### GC × GC-TOF MS: Instrument Setup and Conditions

GC × GC analyses were performed on an Agilent 7890B GC unit (Agilent Technologies, Wilmington DE) coupled to a Markes BenchTOF-Select mass spectrometer featuring tandem ionization (Markes International, Llantrisant, U.K.). The GC transfer line was set at 270 °C. TOF MS tuning parameters were set for single ionization at 70 eV, and the scan range was set at 40–350 *m*/*z* with a spectra acquisition frequency of 100 Hz. The system was equipped with a two-stage KT 2004 loop-type thermal modulator (Zoex Corporation, Houston, TX) cooled with liquid nitrogen and controlled by Optimode v2.0 (SRA Intruments, Cernusco sul Naviglio, Milan, Italy). Modulation period (*P*_M_) and hot jet pulse times were set, respectively, at 3.5 s and 300 ms, with a cold jet stream at the mass flow controller (MFC) from 40 to 8% of the total flow along the run duration. No secondary oven was adopted in the GC × GC setup.

### GC × GC Columns and Settings

The column set was configured as follows: ^1^D DB-HeavyWax column (100% polyethylene glycol; 30 m × 0.25 mm *d*_c_ × 0.25 μm *d*_f_) from Agilent J&W (Wilmington, DE) coupled with a ^2^D OV1701 column (86% polydimethylsiloxane, 7% phenyl, 7% cyanopropyl; 1 m × 0.1 mm *d*_c_ × 0.10 μm *d*_f_) from Agilent Technologies (Wilmington, DE). A fused silica capillary loop (1.0 m × 0.1 mm *d*_c_) was used in the modulator slit.

The GC split/splitless injector port was kept at 250 °C and operated in the split mode with a split ratio of 1:20. The carrier gas was helium at a constant nominal flow of 1.3 mL min^–1^. The oven temperature programming was set as follows: from 40 °C (2 min) to 240 °C (10 min) at 3.5 °C min^–1^.

The *n*-alkane solution for *I*^T^ determination was analyzed under the following conditions: split/splitless injector in the split mode, split ratio 1:50, injector temperature 250 °C, and injection volume 1 μL.

### Combined Untargeted and Targeted (UT) Fingerprinting Workflow

The data processing workflow was designed to comprehensively capture the chemical signature of volatiles from EVOO samples by computing both peak and peak-region features from untargeted (unknowns) and targeted components located over the 2D space. The approach, named UT fingerprinting, was designed on EVOO volatile patterns and further adapted to compositional peculiarities of samples in other fields.^11^ In this study, the targeting (i.e., identification) of analytes was done as the last step of the process after chromatogram realignment over reliable peaks from untargeted components/features.

The generation of untargeted features (i.e., peaks and peak regions) and their realignment across all sample chromatograms were performed by template matching^26^ and actively uses metadata, collected for 2D peaks and peak regions (i.e., retention times, MS spectrum, and detector response) above a signal-to-noise (S/N) threshold value of 100,^23^ to establish correspondences across 2D patterns. Realignment specificity is done by active constraints on MS similarity [i.e., a threshold value of 750 for direct match factor DMF and reverse match factor RMF according to the NIST MS Search algorithm, ver. 2.0 (National Institute of Standards and Technology, Gaithersburg, MD)] between template (reference) and candidate (analyzed) “peak spectra”.^23,27−29^

The chromatographic fingerprinting was performed automatically by the GC Image Investigator V2.9 (GC Image LLC, Lincoln NE) on a random selection of sample chromatograms (*n* = 25) acquired across a time-frame of 2 weeks. It aligned the 25 chromatograms through reliable peaks for registration and generated a composite chromatogram over which peak-region features were delineated and extracted to form a feature template for further processing. Reliable peaks in this study were those that positively matched across all but one of the selected 25 chromatograms (i.e., most constrained condition option).

**Figure 1:**
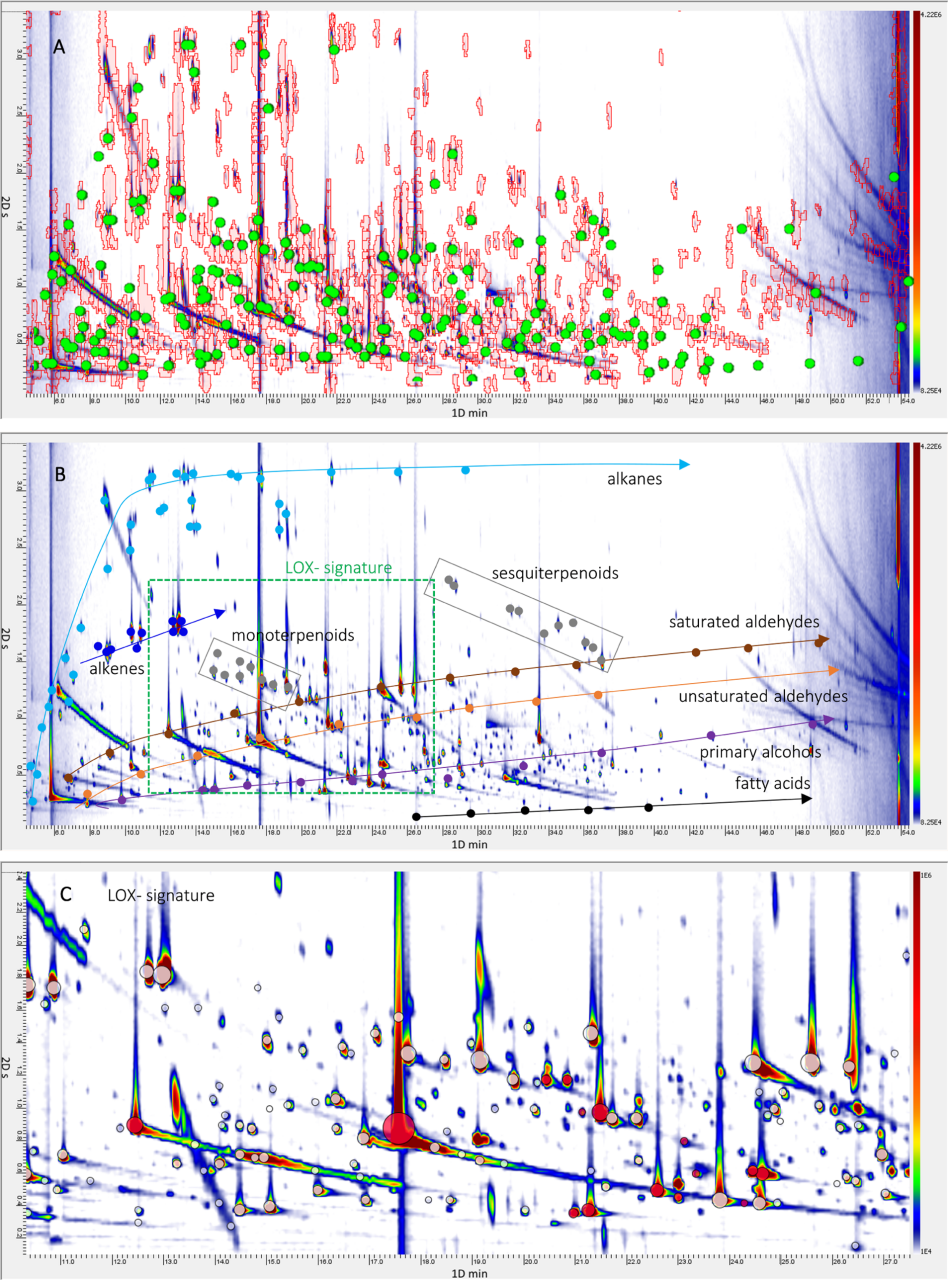
Pseudocolor image (A) of a Sicilian EVOO (Sicilia origin—ID#S1) volatile fraction comprehensively mapped through untargeted and targeted (UT) peak regions (red graphics); identified/targeted analytes (i.e., targeted compounds) are highlighted by green circles. (B) Patterns of analytes, following a retention logic based on the relative retention exerted by the polar × semipolar column combination adopted (alkanes, cyano; alkenes, blue; saturated, brown aldehydes; unsaturated, orange aldehydes; alcohols, purple; terpenoids, gray; and fatty acids, black), are highlighted. (C) Enlarged area of lipoxygenase (LOX) derivatives (red circles).

The resulting feature template includes untargeted (reliable) peaks and peak regions comprehensively capturing the chemical composition of samples. Figure 1A shows the pseudocolor image of a Sicilian EVOO (#S1) overlaid with 591 peak regions (red graphics) and 159 targeted peaks (green circles). Targeting of informative compounds, including EVOO key-aroma compounds, ripening indicators, and potent odorants responsible for coded defects,^30^ was performed at the end of the realignment process over the entire set of chromatograms (*n* = 73). Identifications were confirmed by authentic standards when available in the authors’ laboratory (criterion “a” in Table 1) or by spectral similarity DMF ≥ 900, RMF ≥ 950, and *I*^T^ tolerance ± 20 units (criterion “b”, corresponding to tentative identification in Table 1). Table 1 lists target analytes with ^1^D and ^2^D retention times (^1^*t*_R_; ^2^*t*_R_), precision data (see the Method Performance Parameters section), experimental (Exp.) and tabulated (Lit.) ^1^D *I*^T^ values, and odor descriptors as reported in the reference literature.

**Table 1:** List of 159 Target Peaks, Together with Retention Times in the Two Analytical Dimensions (^1^*t*_R_, ^2^*t*_R_), % Relative Standard Deviation (% RSD) Calculated over Six Analytical Replicates over 2 Weeks and Referred to Retention Times and 2D Peak Volumes; Experimental ^1^D *I*^T^ and Tabulated *I*^T^ Values; Identification Criteria (a) Reference Compound Confirmation or (b) Spectral Direct Match Similarity and *I*^T^ ± 20—Tentative Identification^a^

chemical class	compound name	^1^*t*_R_ (min)	^1^*t*_R_ % RSD	^2^*t*_R_ (s)	^2^*t*_R_ % RSD	2D peak volumes % RSD	exp *I*^T^	Lit *I*^T^	identification
alcohols	2-propanol	7.64	0.39	0.26	1.33	5.37	907	912	a
	2-methyl-1-propanol	12.48	0.76	0.34	0.79	14.20	1064	1081	a
	1-butanol	14.41	0.87	0.36	2.75	13.89	1116	1124	a
	1-penten-3-ol	15.05	0.73	0.36	1.71	6.94	1133	1139	a
	3-methyl-1-butanol	16.92	0.79	0.40	0.88	6.94	1180	1184	b
	1-pentanol	18.08	0.75	0.48	2.91	20.83	1210	1216	a
	(*Z*)-2-penten-1-ol	20.94	0.50	0.34	0.92	15.22	1282	1289	a
	(*E*)-2-penten-1-ol	21.29	0.72	0.34	4.50	10.55	1291	1296	a
	(*Z*)-3-hexen-1-ol	22.58	0.99	0.46	3.54	4.05	1324	1344	a
	1-hexanol	22.98	0.69	0.50	1.37	4.72	1335	1338	a
	(*Z*)-2-hexen-1-ol	23.74	0.74	0.42	3.78	6.08	1355	1375	a
	(*E*)-2-hexen-1-ol	24.21	0.00	0.70	3.85	4.66	1367	1379	a
	1-butoxy-2-propanol	24.56	0.29	0.40	3.60	2.77	1376	1363	b
	1-heptanol	26.43	0.88	0.52	2.60	14.77	1425	1423	a
	1-octen-3-ol	26.72	0.91	0.56	0.64	2.47	1433	1437	a
	2-ethyl-1-hexanol	27.77	0.20	0.60	2.12	3.32	1462	1470	a
	1-octanol	29.75	0.23	0.66	0.86	13.60	1517	1518	a
	1-nonanol	33.02	0.55	0.66	1.59	14.41	1610	1616	a
	4-butoxy-1-butanol	34.83	0.04	0.54	1.21	2.64	1664	1668	b
	1-decanol	36.98	0.87	0.70	3.16	4.15	1730	1738	a
	2-(2-butoxyethoxy)-ethanol	37.92	0.94	0.52	0.93	19.81	1759	1364	b
	benzyl alcohol	40.37	0.87	0.28	3.79	14.78	1839	1846	a
	1,4-butanediol	41.30	0.83	0.28	0.71	5.05	1867	1861	b
	phenylethyl alcohol	41.48	0.66	0.34	0.10	2.45	1875	1877	a
	1-dodecanol	42.88	0.08	0.68	0.77	9.55	1921	1924	a
	phenol	43.93	0.77	1.60	3.59	12.40	1956	1957	a
	1-tetradecanol	47.56	0.48	0.65	2.55	11.68	2118	2137	b
	2-phenoxyethanol	47.83	0.29	1.58	4.54	15.91	2130	2145	b
	1-hexadecanol	54.25	0.34	1.06	3.01	4.02	2345	2356	b
esters	ethyl acetate	6.88	0.35	0.42	3.94	13.34	866	875	a
	2,2-dimethylpropanoate	7.70	0.58	0.72	2.18	16.23	909	913	a
	butyl acetate	12.13	0.73	0.84	3.12	14.29	1055	1064	a
	isoamyl acetate	13.94	0.91	1.02	0.85	18.09	1104	1109	a
	2-methylpropyl butanoate	14.82	0.01	1.08	1.39	7.43	1127	1139	b
	butyl isobutyrate	14.99	0.28	1.36	1.11	15.72	1131	1140	b
	butanoic butanoate	17.73	0.43	1.28	4.96	19.18	1201	1212	a
	3-hydroxy-2-butanone	20.01	0.84	0.34	2.91	12.18	1259	1270	a
	hexyl acetate	20.42	0.81	1.14	3.31	19.31	1269	1275	a
	(*Z*)-3-hexenyl acetate	21.47	0.49	0.92	3.33	19.79	1296	1310	a
	butyl 2-ethylhexanoate	27.42	0.54	1.88	4.55	6.49	1452	1459	a
	methyl benzoate	30.67	0.59	0.58	3.50	10.67	1546	1560	a
	ethyl benzoate	32.55	0.40	0.72	4.92	3.38	1597	1612	a
	methyl salicylate	37.57	0.00	0.56	2.37	3.99	1748	1755	b
	butyl benzoate	40.31	0.79	0.82	1.30	20.29	1837	1846	a
lactones	4-hydroxy-2-hexenoic acid lactone	31.50	0.35	0.46	3.27	15.41	1567		b
	butyrolactone	32.20	0.72	0.42	2.15	20.63	1587	1601	a
	β-angelica lactone	34.24	0.15	0.42	1.45	15.98	1647	1664	b
	δ-pentalactone	35.12	0.59	0.52	0.56	3.22	1672	1684	a
	λ-hexalactone	38.09	0.35	0.60	3.51	5.84	1765		a
fatty acids	acetic acid	26.43	0.13	0.12	3.94	15.78	1425	1427	a
	propanoic acid	29.52	0.23	0.14	3.99	18.99	1510	1516	a
	butanoic acid	31.97	0.35	0.62	3.17	20.39	1580	1581	a
	pentanoic acid	36.05	0.96	0.30	0.24	5.53	1700	1704	a
	hexanoic acid	39.55	0.15	0.20	0.13	13.26	1812	1817	a
	heptanoic acid	42.64	0.23	0.26	4.87	14.40	1914	1920	a
	octanoic acid	45.73	0.04	0.26	4.27	5.55	2053	2058	a
	nonanoic acid	48.59	0.02	0.42	1.11	10.84	2181	2180	a
	decanoic acid	51.63	0.54	0.30	4.35	16.35	2239	2240	a
hydrocarbons	*n*-hexane	3.91	0.40	0.34	1.50	10.88	600		a
	cyclopentane	4.20	0.95	0.22	2.12	3.19	631		b
	2,4-dimethylhexane	4.38	0.39	0.54	0.92	10.70	649		b
	1,4-pentadiene	4.61	0.79	0.26	2.53	16.93	675		b
	cyclohexane	4.96	0.92	0.50	0.31	14.37	700	719	a
	*n*-heptane	4.96	0.16	0.72	4.22	15.01	700		a
	2-methylheptane	5.31	0.27	0.88	3.15	6.70	754		b
	*n*-octane	5.72	0.99	1.06	2.15	15.96	800		a
	1-octene	6.24	0.95	1.00	0.84	5.71	822	838	b
	2,3-dimethylheptane	6.48	0.64	1.64	0.57	9.44	839	847	b
	2,4-dimethyl-1-heptene	6.77	0.83	1.12	2.12	18.67	859	878	b
	*n*-nonane	7.62	0.33	1.72	3.00	4.08	900		a
	benzene	8.11	0.37	0.48	1.61	15.84	924	934	a
	3,4-diethyl-1,5-hexadiene (meso)	8.87	0.50	1.54	3.34	6.90	952	966	a
	3,4-diethyl-1,5-hexadiene (RS + SR)	9.10	0.59	1.52	1.50	15.25	961	968	a
	*n*-decane	10.21	0.97	2.44	1.90	5.35	1000		a
	(5*Z*)-3-ethyl-1,5-octadiene	10.33	0.62	1.70	4.40	18.95	1005	1006	a
	(5*E*)-3-ethyl-1,5-octadiene	10.79	0.04	1.72	4.27	13.16	1018	1012	a
	4-methyldecane	10.79	0.89	2.72	0.23	10.79	1018	1022	b
	toluene	11.03	0.54	0.68	4.86	13.14	1024	1024	a
	1-decene	11.43	0.88	2.04	0.73	20.43	1035	1039	b
	(*E*,*Z*)-3,7-decadiene	12.66	0.36	1.82	0.49	19.42	1069	1069	a
	(*E*,*E*)-3,7-decadiene	12.95	0.87	1.80	1.42	6.64	1077	1077	a
	*n*-undecane	13.77	0.44	2.86	1.99	10.91	1100		a
	1,3-dimethylbenzene	14.12	0.69	0.86	0.50	14.17	1109	1122	b
	ethylbenzene	14.47	0.62	0.86	4.45	11.26	1118	1125	b
	1-dodecene	17.56	0.62	2.50	0.89	19.10	1197	1192	b
	*n*-dodecane	17.68	0.22	3.04	0.89	2.11	1200		a
	Styrene	19.13	0.56	0.66	0.29	8.42	1237	1242	b
	(*E*)-4,8-dimethylnona-1,3,7-triene	20.07	0.59	1.46	4.56	8.14	1260	1266	b
	1,2,3-trimethylbenzene	20.18	0.78	0.96	1.11	13.72	1263	1282	b
	n-tridecane	21.64	0.90	3.08	3.31	10.77	1300		a
	1-ethenyl-4-ethylbenzene	26.13	0.51	0.86	4.38	13.83	1417		b
terpenoids	α-pinene	10.68	0.88	1.62	1.26	17.60	1015	1017	a
	β-pinene	13.07	0.84	1.60	2.12	3.80	1081	1072	a
	ß-myrcene	15.69	0.54	1.32	2.11	15.33	1149	1154	a
	limonene	17.09	0.68	1.40	4.56	18.55	1185	1190	a
	eucalyptol	17.38	0.59	1.54	1.17	6.53	1192	1195	a
	terpinene	18.43	0.81	1.24	2.99	12.14	1219	1221	a
	(*E*)-ß-ocimene	19.13	0.79	1.24	0.58	11.92	1237	1239	a
	α-copaene	28.35	0.70	2.16	1.24	17.55	1478	1485	a
	linalool	29.40	0.78	0.90	1.21	8.86	1507	1507	a
	α-muurolene	36.40	0.92	1.54	1.43	17.84	1711	1714	b
	α-farnesene	36.98	0.16	1.44	1.30	10.91	1730	1740	a
saturated aldehydes	propanal	5.43	0.05	0.28	1.73	4.23	769	762	a
	butanal	6.71	0.43	0.42	2.09	14.68	856	861	a
	pentanal	9.10	0.01	0.64	2.06	19.63	961	965	a
	hexanal	12.43	0.40	0.86	4.41	14.35	1063	1066	a
	heptanal	15.87	0.55	1.02	1.18	20.24	1154	1161	a
	2-ethylhexanal	16.45	0.74	1.32	0.89	18.25	1169	1187	a
	octanal	19.78	0.19	1.14	2.02	14.25	1253	1268	a
	nonanal	24.44	0.39	1.22	1.24	20.93	1373	1380	a
	decanal	28.35	0.09	1.30	3.84	5.74	1478	1475	a
	undecanal	32.08	0.57	1.34	3.93	12.16	1583	1585	a
	dodecanal	35.64	0.78	1.40	2.65	14.35	1688	1688	a
	tridecanal	38.79	0.74	1.42	1.18	7.32	1787	1792	b
unsaturated/aromatic aldehydes	(*E*)-2-butenal	10.27	1.00	0.90	0.66	14.45	1003	1002a	a
	(*Z*)-2-pentenal	13.24	0.01	0.64	4.81	13.17	1085	1101	a
	(*E*)-2-pentenal	14.06	0.96	0.64	2.76	13.05	1107	1111	a
	3-methyl-2-butenal	16.16	0.67	0.58	0.61	19.81	1161	1164	a
	(*Z*)-2-hexenal	16.86	0.13	0.78	3.32	14.98	1179	1183	a
	(*E*)-2-hexenal	17.50	0.80	0.82	1.59	2.06	1195	1204	a
	(*Z*)-2-heptenal	18.32	0.96	0.72	3.02	8.08	1216	1218	a
	2-ethyl-2-hexenal	20.83	0.45	1.14	1.94	13.83	1279	1285	a
	(*E*)-2-heptenal	21.93	0.51	1.06	0.69	15.83	1307	1318	a
	(*E*,*Z*)-2,4-hexadienal	24.38	0.29	0.58	4.48	16.10	1371	1373	a
	(*E*,*E*)-2,4-hexadienal	24.50	0.38	0.61	2.32	12.38	1375	1376	a
	(*E*)-2-octenal	24.97	0.29	0.86	1.01	19.78	1386	1391	a
	(*E*,*E*)-2,4-heptadienal	26.89	0.83	0.68	0.96	15.59	1438	1441	a
	benzaldehyde	29.05	0.16	0.50	3.46	10.58	1497	1499	a
	(*E*)-2-nonenal	29.28	0.76	1.10	4.60	17.09	1503	1509	a
	(*E*)-2-decenal	33.43	0.44	1.36	3.20	13.67	1622	1625	a
	(*E*,*E*)-2,4-decadienal	37.22	0.74	0.90	1.98	3.81	1737	1740	a
	2,4-dimethylbenzaldehyde	38.68	0.21	0.64	4.59	4.09	1783	1789	b
ketones	acetone	5.72	0.08	0.30	4.97	14.82	800	819	a
	2-butanone	7.12	0.83	0.44	1.48	10.16	880	887	a
	3-buten-2-one	8.17	0.40	0.40	2.16	13.95	926	931	a
	2,3-butanedione	8.75	0.27	0.38	0.41	13.05	948	954	a
	1-penten-3-one	10.27	0.27	0.56	2.38	6.61	1003	1019	a
	2,3-pentanedione	10.97	0.14	0.52	2.12	14.05	1023	1026	a
	3-penten-2-one	13.88	0.63	0.58	2.75	19.70	1103	1106	a
	4-heptanone	14.06	0.47	1.10	0.25	19.18	1107	1118	b
	3-heptanone	15.11	0.26	1.08	4.68	9.32	1134	1141	a
	2-heptanone	16.33	0.27	1.02	2.66	5.21	1166	1169	a
	2-octanone	19.22	0.45	1.10	1.97	13.31	1239	1244	a
	6-methyl-5-hepten-2-one	21.70	0.11	0.90	3.33	18.52	1302	1313	a
	(*E*)-3-octen-2-one	24.68	0.69	0.92	4.03	12.79	1379	1384	a
	5-methyl-2-(1-methylethyl)-cyclohexanone	27.13	0.07	1.30	2.64	11.88	1444	1448	b
	3,5-octadien-2-one	28.88	0.24	0.76	0.14	10.74	1492	1492	a
	2-decanone	29.46	0.38	1.54	2.53	11.03	1508	1515	a
others	tetrahydrofuran	6.48	0.29	0.46	3.65	16.69	838	845	b
	1-methoxyhexane	8.46	0.86	1.12	0.81	11.63	937	941	b
	2-ethylfurane	8.46	0.03	0.54	3.58	2.11	937	944	a
	acetonitrile	9.74	0.75	0.26	2.91	10.67	985	988	b
	furfural	28.18	0.84	0.40	3.16	4.56	1473	1477	a
	dimethyl sulfoxide	30.28	0.13	0.38	0.19	3.85	1532	1549	b
	1-chloro dodecane	34.48	0.98	1.76	3.71	20.48	1653	1661	b
	3,4-dimethyl-2,5-furandione	35.41	0.52	0.66	4.77	2.66	1681	1685	b
	acetamide	36.46	0.01	0.18	3.30	9.61	1713	1725	b
	phthalide	53.43	0.66	1.76	3.84	17.87	2312	2323	a
	diethyl phthalate (IS dilution solvent)	53.84	0.07	0.62	0.12	18.26	2329	2332	a
	**average % RSD value**		**0.34**		**3.01**	**11.98**			

a Odor descriptors as reported in the reference literature.^17,19,36,37,44^

The output table collecting 2D peaks and peak regions aligned across all chromatograms with feature-related metadata (^1^D and ^2^D retention times, MS spectrum, base peak and molecular ion *m*/*z*, and TIC response) was stored and made available for further processing.

Supporting Information Table S2 lists untargeted and targeted peak-region features included in the UT template, together with their experimental ^1^D *I*^T^ values, retention times in the two analytical dimensions (^1^*t*_R_, ^2^*t*_R_), % relative standard deviation (% RSD) on retention times across all analyses, and reference MS spectral signature from the peak-apex spectrum.

### Method Performance Parameters

Repeatability was evaluated on analytical descriptors considered fundamental for an accurate chromatographic fingerprinting based on both 2D peak patterns and analyte responses. Therefore, % RSD was calculated on retention times and analyte % response (% normalized 2D volumes over IS) for all targeted compounds and on analytical replicates of the same sample analyzed every 2 days over the 2 weeks of the study (*n* = 6). Results are reported in Table 1. Mean % RSDs on retention times were 0.34% for ^1^D (^1^*t*_R_) and 3.01% for ^2^D (^2^*t*_R_). Maximum % RSD on percent response was instead 20.93%, reported for nonanal, while the mean value was 11.98%.

### Data Acquisition and 2D Data Processing

Data were acquired by TOF-DS software (Markes International, Llantrisant, U.K.) and processed by the GC Image V2.9 suite (GC Image, LLC Lincoln, NE).

The data files of peak-region features from each chromatogram were exported in the “.xls” format (Microsoft Excel) and then converted to the MATLAB format (version R2017b). All of the multivariate analyses were performed using PLS_Toolbox 8.6.1 (Eigenvector Research, Manson, WA) for the MATLAB environment (MathWorks Inc., Massachusetts, R2017b). Principal component analysis (PCA), partial least-squares regression (PLS), and soft independent modeling for class analogy (SIMCA) were applied as exploratory analysis, variable selection, and classification method, respectively. In addition, data were preprocessed by autoscaling before model development. Microsoft Excel spreadsheet was used for similarity analysis.

## Results and Discussion

Chromatographic fingerprinting based on comprehensive two-dimensional separations has a great potential for discrimination and identification of samples based on their chemical signatures, a process described as identitation.^10^ Moreover, it offers further advantages when mass spectrometry is used at the detection level, providing additional information for analyte putative identification. This step gives access to a higher information level on sample properties and characteristics.^7,18,31^

The strategy adopted to decrypt the hidden information from volatile patterns of EVOOs harvested in different Italian regions collects information from untargeted and targeted (UT) features. It is a fingerprinting approach designed to comprehensively map all detectable volatiles from GC × GC-TOF MS analyses.^16^ Chromatogram processing was done by a validated workflow described in the Combined Untargeted and Targeted (UT) Fingerprinting Workflow section; the output was a data matrix of dimensions 73 × 519 (i.e., samples × features) with a subset of 159 identified (targeted) compounds.

The next section highlights the fundamental role of high-resolution separations and retention pattern logic based on effective identitation of samples. Machine learning, based on multivariate statistics and modeling algorithms, will be presented as a key tool to access a higher level of information to identify distinctive regional marker patterns.

### Complex and Multidimensional EVOO Volatilome

EVOO is highly appreciated by consumers because of its unique and characteristic flavor, which reflects the chemical complexity and dimensionality^32^ of its volatile fraction, characterized by the presence of many compounds, especially carbonyls (e.g., aldehydes, ketones), esters, alcohols, and hydrocarbons (e.g., linear, aromatic, terpenoids, etc.). Odor-active compounds, with a low odor perception threshold, and volatiles lacking sensory features (i.e., interferents),^33^ concur in the modulation of the “odor code” while triggering aroma perception, whose objectification by instrumental methods is challenging.^9,34^ However, EVOO volatiles encrypt additional information about relevant functional variables including olive cultivars, the olive tree’s harvest region and local pedoclimatic conditions, olive ripeness, technological processes, and storage condition.^1,2,35,36^

Figure 1A shows the pseudocolor image of a Sicilian EVOO (Sicilia origin—#S1) volatile fraction comprehensively mapped through untargeted and targeted (UT) peak regions (red graphics); identified/targeted analytes (i.e., targeted compounds) are highlighted by green circles. Patterns of analytes, following a retention logic based on the relative retention exerted by the polar × semipolar column combination adopted, are highlighted in Figure 1B,C.

Compounds derived from oxidative cleavage of linoleic and linolenic acids, promoted by lipoxygenase (LOX) and hydroperoxide lyase (HPL) pathways, constitute the LOX signature (green-color area in Figure 1B and enlarged area in Figure 1C), which is the most abundant fraction in high-quality EVOOs.^1,13^ It is characterized by the presence of C_6_ and C_5_ compounds, in particular aldehydes, alcohols, ketones, and esters (e.g., hexanal, (*E*)-2-hexenal, 1-penten-3-ol, 1-hexanol, 1-penten-3-one, hexyl acetate, etc.), fundamental to define positive attributes as fruity and green.^14,21^

Saturated and unsaturated aldehydes (respectively, in brown and orange) are mainly produced by oxidation of unsaturated fatty acids.^37^ While C_6_ and C_5_ unsaturated aldehydes from LOX are correlated to positive attributes, the others, with a higher molecular weight and low odor threshold (e.g., (*E*)-2-heptenal, (*E*)-2-octenal, (*E*)-2-decenal, heptanal, octanal, and nonanal), are indicated in many studies as responsible for the rancid off-flavor with unpleasant and penetrating notes.^19,37−39^ Alcohols (purple line in Figure 1B), represented by 30 congeners here identified, have a strong retention in the ^1^D polar column and are well separated by informative carbonyls. Of them, the most relevant are 1-octen-3-ol, 1-nonanol, and 1-decanol because of their decisive role in defining sensory defects eliciting fatty, rancid, earthy, and mushroom-like notes.^24,36^

Short-chain fatty acids (Figure 1B black line) derive from the oxidation of the corresponding aldehydes^19,37^ with propanoic and butanoic acids as the most odor-active, followed by pentanoic and heptanoic acids. Their presence was correlated to the perception of rancid and fusty defects.^1,37,38^

Hydrocarbons (Figure 1B in cyano) have a negligible contribution in the definition of the EVOO flavor, although some unsaturated derivatives (i.e., 3-ethyl-1,5-octadiene and 4,8-dimethyl-1,3,7-nonatriene) were linked to green and fruity notes^13^ or to rancid and fishy aroma.^1^ Moreover, a series of C10 alkenes, i.e., 3,4-diethyl-1,5-hexadiene (*RS* or *SR*), 3,4-diethyl-1,5-hexadiene (meso), (5*Z*)-3-ethyl-1,5-octadiene, (5*E*)-3-ethyl-1,5-octadiene, (*E*,*Z*)-3,7-decadiene, and (*E*,*E*)-3,7-decadiene, whose elution region is highlighted in blue in Figure 1B, are known to be diagnostic markers of early ripening stages of olives (Angerosa, Camera, D’Alessandro, & Mellerio, 1998), while *n*-octane is an indicator of over-ripening.^3,16,24^

The presence and abundance of terpenes (gray rectangles in Figure 1B) are of particular interest because of their role as indicators of geographical origin^7,14^ or of ripening, e.g., α-farnesene.^3^ Moreover, they contribute to defining positive attributes, such as wood, lemon, and roselike odors.^13,40^

Lactones are generally detected in low but variable amounts in EVOO, and their relative concentration is cultivar-specific.^36^ Esters as well, closely eluting to lactones, contribute to defining fruity notes, with C_6_ and C_5_ derivatives deriving from the LOX pathway that dominates the class.^1,21,36^

### Multivariate Analysis

**Figure 2:**
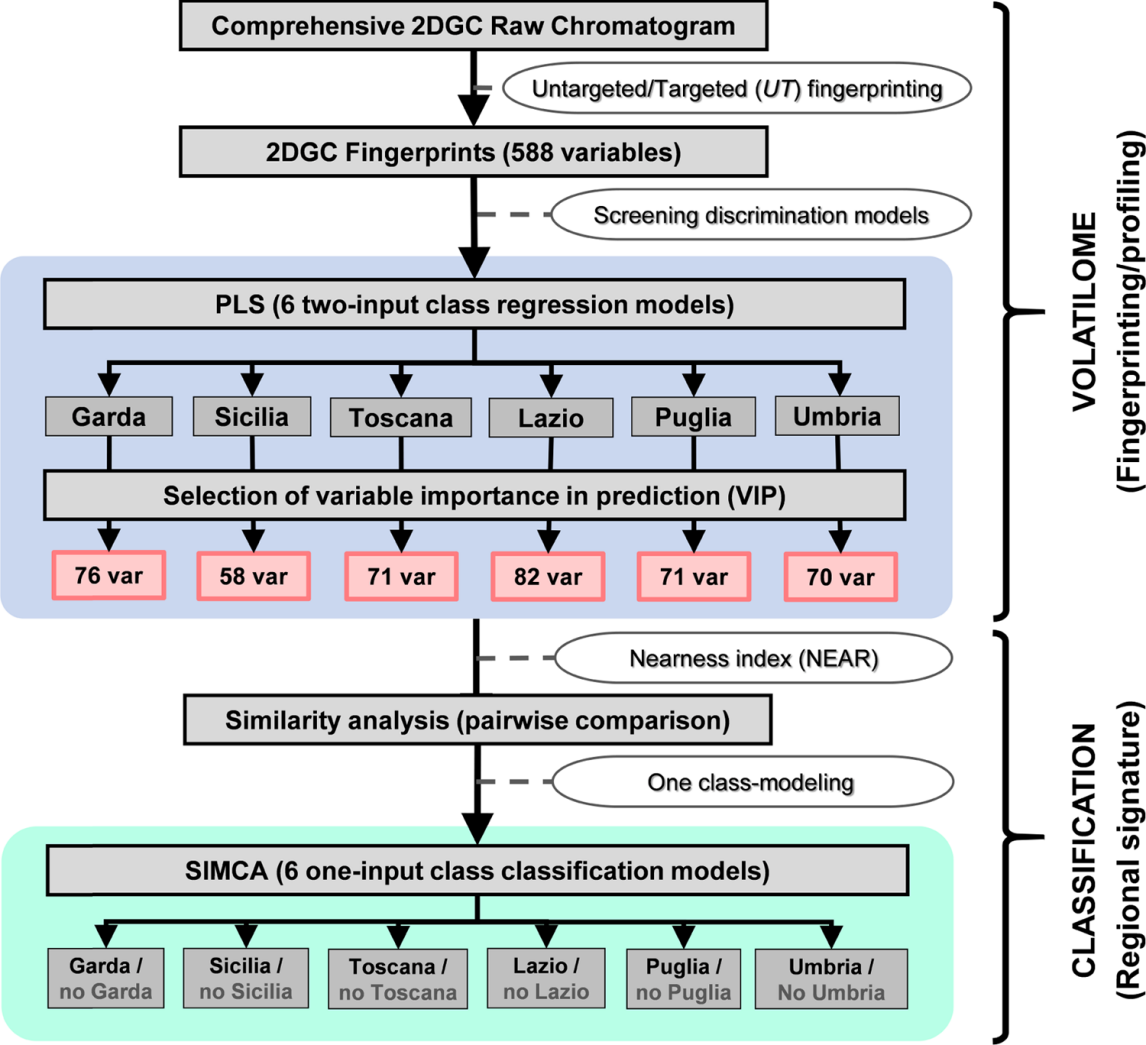
Workflow including data processing (i.e., fingerprinting and profiling) and machine learning.

First, an exploratory unsupervised analysis was carried out applying PCA; data structure was examined to check whether geographical-origin-related intrinsic groupings of olive oil samples were detectable. Then, six two-level PLS regression models (one for each concerned Italian region) were built to obtain the variable importance in prediction (VIP) scores and to select the variables that contribute the most to characterize each EVOO belonging to a particular Italian region against the rest of the samples. From these PLS models, the six characteristic volatile patterns, one for each geographical region, were delineated and a similarity analysis of the characteristic pattern of each region was carried out by applying the nearness index. Finally, six one-input class SIMCA classification models were developed and validated. Figure 2 shows the multivariate analysis workflow designed to capture informative and diagnostic patterns capable of correctly classifying/discriminating EVOO production regions.

### Exploratory Analysis

PCA was initially performed considering the 591 variables (i.e., peak-region features) per sample (*n* = 73). After inferring from this first PCA model, three variables were removed: phthalide, (*E*)-2-hexenal, and toluene because the related loadings were very large in all cases, and they were masking the behavior of the other variables. Finally, a new PCA with 588 variables was developed and all of the successive multivariate analysis steps were carried out with these variables.

**Figure 3:**
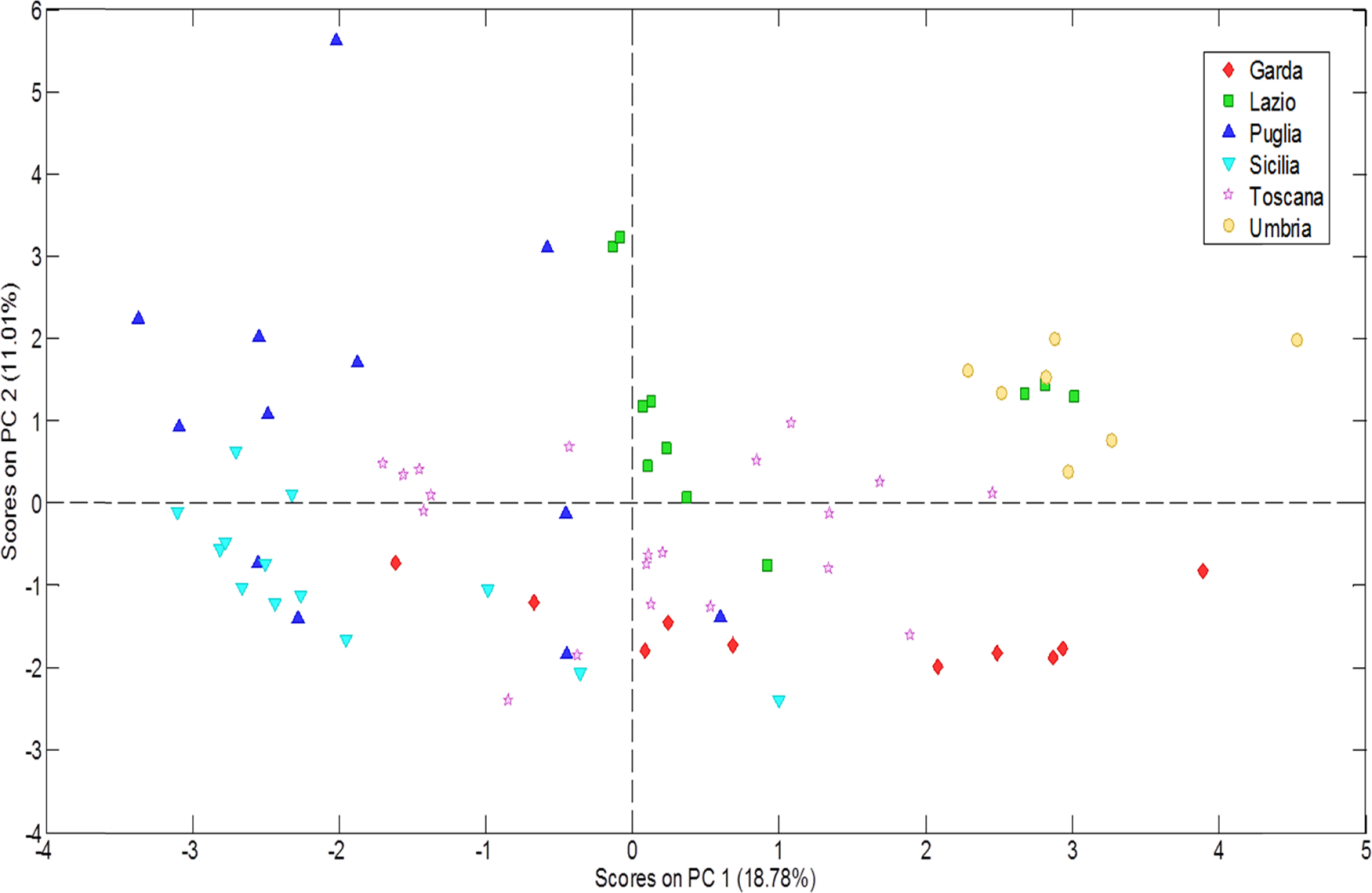
Principal component analysis (PCA) score plot on PC1 vs PC2 accounting for 79.73% of the total explained variance. The PCA model is based on the TIC % response from UT peak regions comprehensively covering the chromatographic space. EVOOs from different Italian regions are displayed in different colors.

The new PCA model was built with 12 principal components, which explained a total variance of 79.73%. Figure 3 displays the score plot on PC1 vs PC2. Some particular grouping trends were observed for the olive oil samples from Sicilia, Lazio, and Umbria. In addition, Garda and Puglia were spread over the bottom and top halves of PC1. Notice that the variance explained by both PCs is approximately 30% of the total variability. This implies that the main source of variability in the data is not related to geographical origin. Nevertheless, it is sufficient to propose classificatory models.

### Variable Selection: Characteristic Profile

The variable importance in the projection (VIP) score, which summarizes the overall contribution of each variable to the PLS model, was used as the variable selection strategy to highlight characteristic volatile patterns for each region. The “greater-than-one-rule” was applied for selecting the VIP scores, and only about 12% of the total number of variables (588) were selected as characteristics. In this way, the number of selected variables per region was Garda, 76; Sicilia, 58; Toscana, 71; Lazio, 82; Puglia, 71; and Umbria, 70; accounting for a total of 121 variables. Table 2 shows the numbers of LVs chosen as well as the percentage of variance explained for each model.

**Table 2:** Characteristics of the PLS Models

model	LVs	% variance
Garda	8	93.72
Sicilia	4	86.80
Toscana	8	94.80
Lazio	8	95.06
Puglia	7	91.46
Umbria	9	95.78

Tables S3–S8 list, for each Italian region, the specific variables and include both untargeted and targeted components.

### Similarity Study

The similarity analysis among the region characteristic patterns was carried out by calculating similarity indices. Such indices are defined as a number between 0 and 1, which describes the equivalence of two objects characterized by multivariate data; the value 0 indicates maximum difference and 1 implies maximum similarity. In this study, the nearness index (NEAR)^41^ was employed; it can be calculated by eq 1
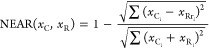
1where *x*_c*_i_*_ and *x*_r*_i_*_ symbolize each element of the considered and reference characteristic profiles, respectively. Note that eq 1 has two terms. The second term is a quotient between the sum of distances between the different elements of the two vectors (global distance) and the value of the sum of these elements. In this way, a normalized global distance between 0 and 1 is calculated. This second term is subtracted from 1 to convert the distance (which measures dissimilarity) into similarity so that 1 represents the total coincidence and 0 represents the null coincidence.

Equation 1 could also be reformulated in the matrix notation as reported in eq 2
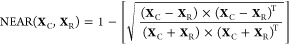
2where, correspondingly, **X**_c*_i_*_ and **X**_R*_i_*_ are the considered and reference characteristic profile vectors, respectively (the superscript T denotes the transposed matrix).

To carry out the similarity study, a new reduced tertiary vector consisting of 0, 1, and 2 codes for each region was built from the regional characteristic profiles; results are reported in Table 3. The following rules were applied to establish the aforementioned codes:

- 0: It was assigned to those variables not selected as part of the regional characteristic pattern, e.g., variable 32 corresponding to methyl benzoate was selected only for the Puglia profile, and thus, this variable was codified with the value 0 for the remaining reduced tertiary vectors.
- 1: It was assigned to those variables whose VIP scores ranged from 0 to 1, e.g., variable 46 corresponding to ethyl benzoate was selected for Lazio, Puglia, and Umbria profiles.
- 2: It was assigned to those variables whose VIP scores were greater than 1, e.g., variable 8 corresponding to α-copaene had a VIP score greater than 1 for Garda, Sicilia, Toscana, Lazio, and Puglia profiles and a VIP score between 0 and 1 for the Umbria profile. Thus, in the five patterns (Garda, Sicilia, Toscana, Lazio, and Puglia), this variable was codified with value 2 and in Umbria having value 1.

**Table 3:**
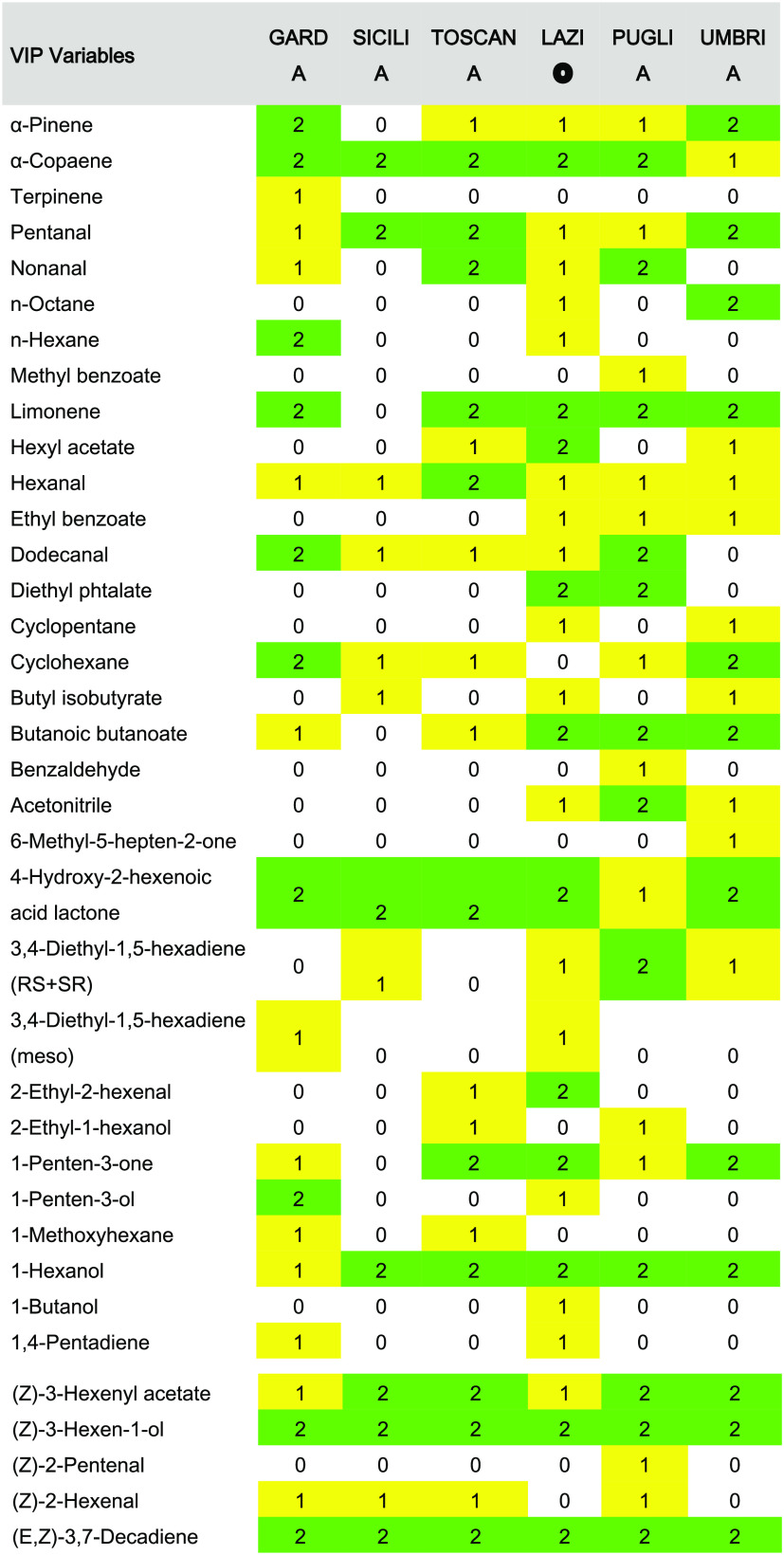

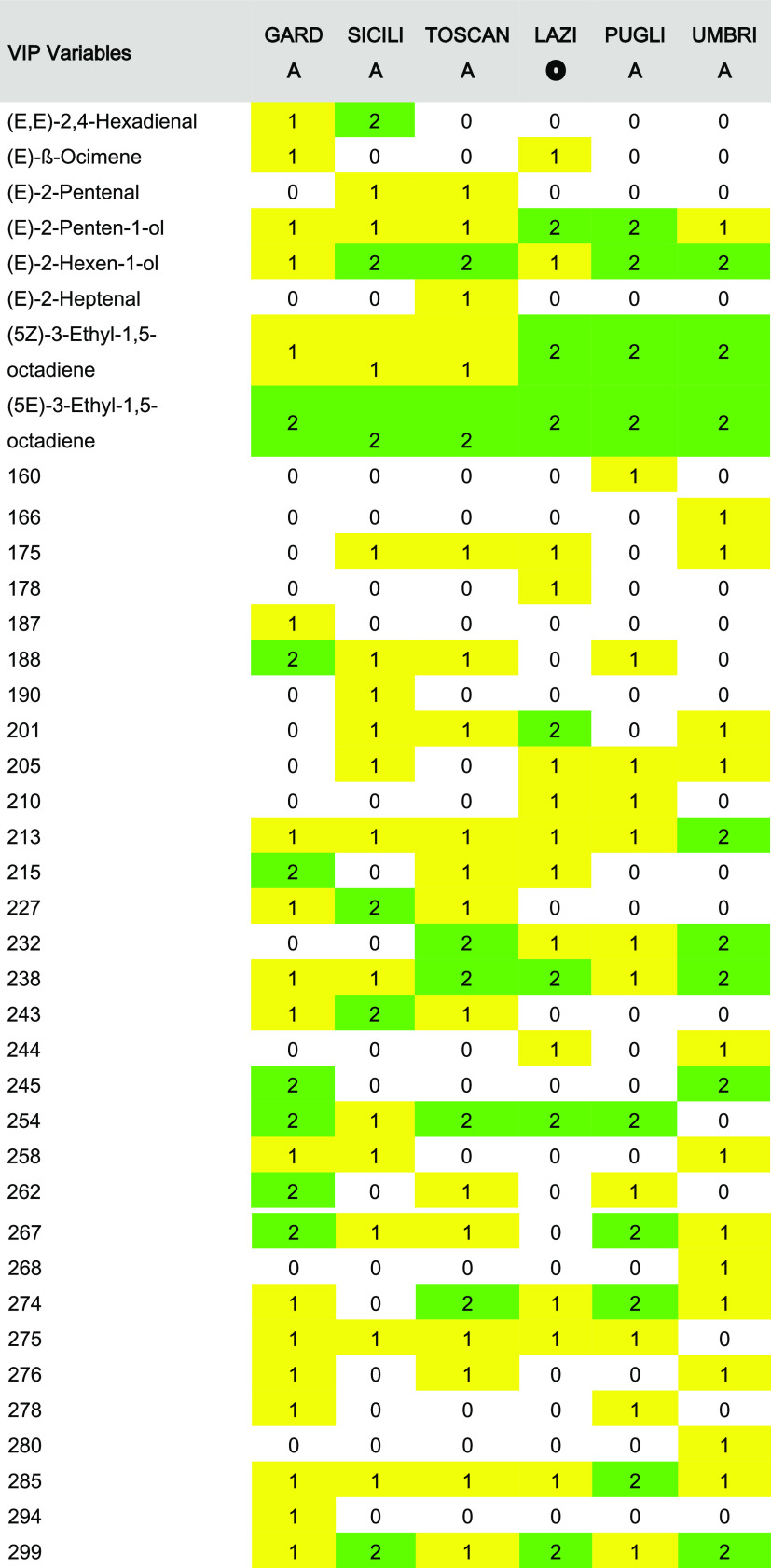

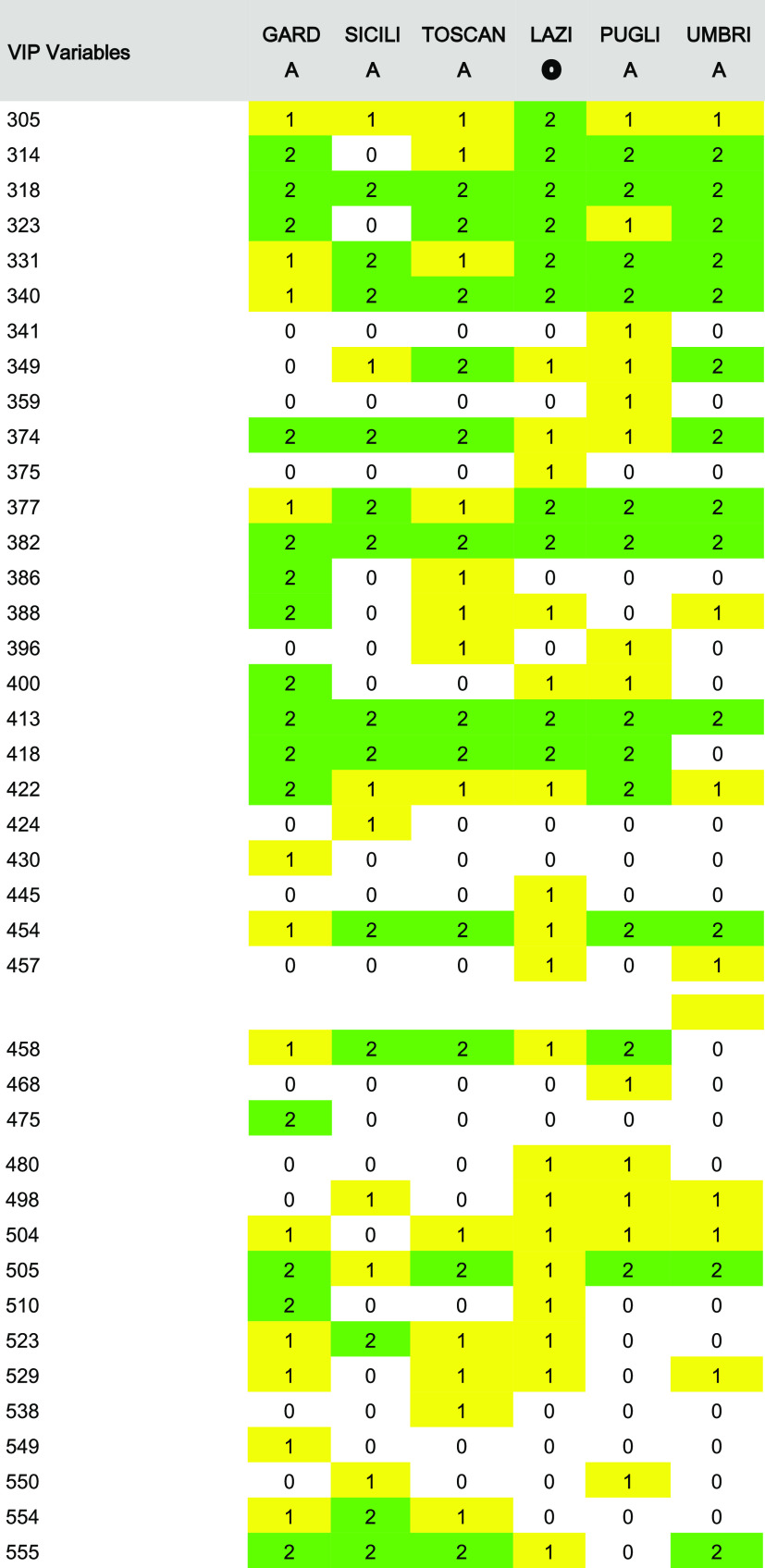

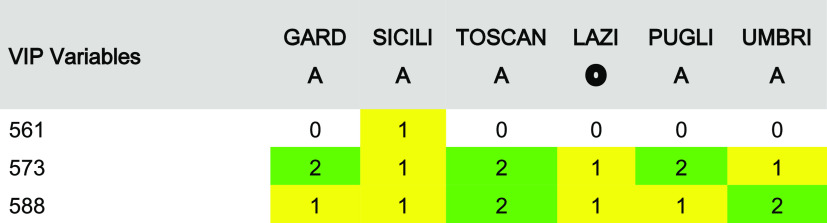
Reduced Tertiary Vector from Six Studied Regions from Italy

**Figure 4:**
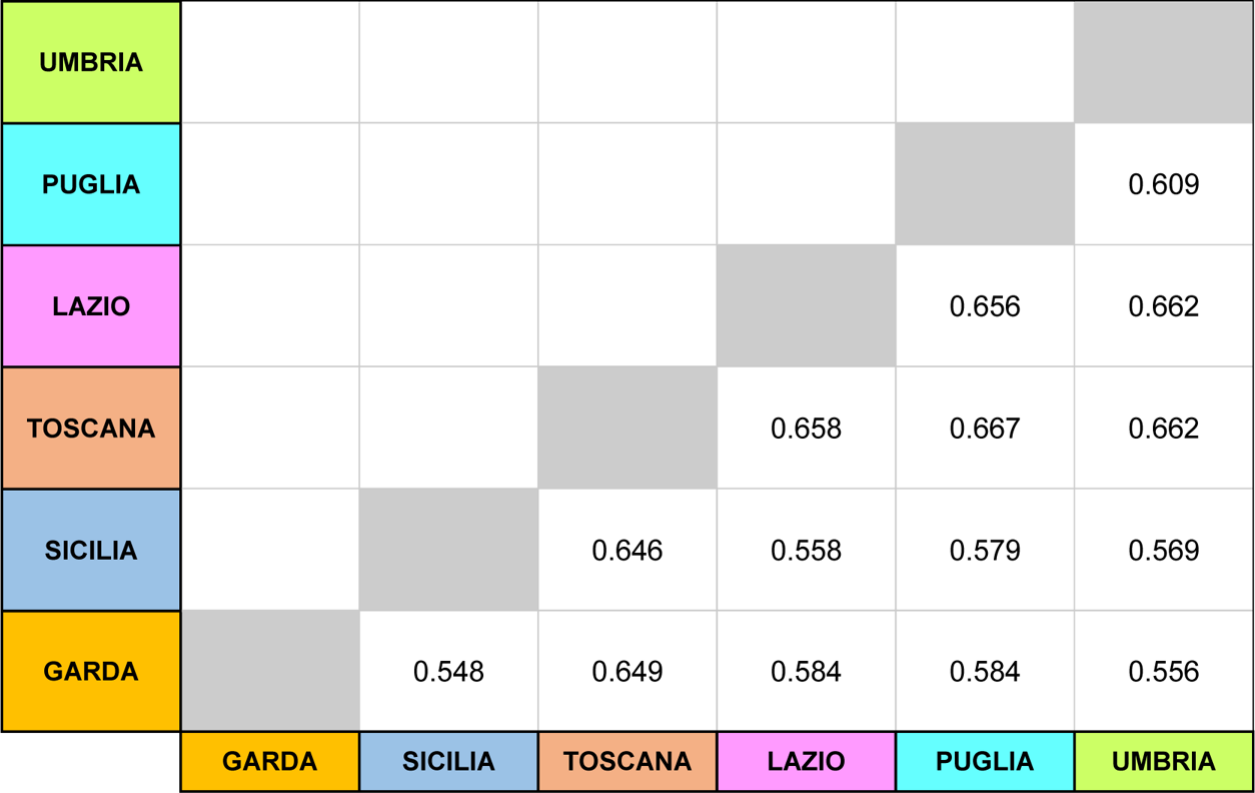
Similarity matrix based on the NEAR index resulting from pairwise comparison of regional patterns.

Once the reduced tertiary vectors from characteristic patterns were pairwise compared, a similarity matrix was constructed from the found NEAR values, which is shown in Figure 4.

As can be seen in the similarity matrix, in all cases, the NEAR value is significantly less than 1, indicating that the volatile profile/pattern between the regions is significantly dissimilar. Therefore, it may be used to classify samples according to geographical origin. In addition, there were five variables out of the 121 selected that were present in all characteristic patterns having a code higher than 1. It was therefore decided to remove them from the classification models as their contribution to the discrimination among regions would not be relevant.

### Classification According to Harvest/Production Region Unique Signature

The most conventional way to develop a classification model is based on building a model with two input classes, the target class and the nontarget class, but a valid alternative is performing the same classification method by training with a single input class, i.e., the target class.^42^ Working with one input class classification has significant advantages in food authentication: the model is trained using the data from representative samples from genuine foods (target class) and no other samples are required. In fact, some authors have stated that it is advisable to develop models using a one-class classifier in the case of food authentication. Indeed, if a well-known discriminant method such as partial least squares-discriminant analysis (PLS-DA) is used and a new sample does not belong to any such class, the discriminant analysis is unable to properly define the belonging of the sample to one particular class. Conversely, a one-class classifier such as SIMCA establishes if the acceptance is around the target class, delimiting the target samples from other classes.^43^

SIMCA involves building a classification method in which each class of training set is modeled independently and the assignment of an unknown sample as belonging to a specific class is based on the nearest distance to the corresponding regions established in the space of principal components. Six one-input class SIMCA models were built, one for each Italian region. Each individual model was developed using the 116 untargeted/targeted features, which were selected in at least one of the regional characteristic patterns. The aim was to generate overall models suitable for application in routine analysis. Otherwise, should it be required to classify a sample of unknown origin, whose characteristic variables would be selected or chosen? In this way, any classification model developed can be applied, and it will be possible to assign a class to the sample. Table 4 shows the numbers of PCs chosen for each model and the samples used in the training and validation steps.

**Table 4:** Characteristics of the SIMCA Models

origin	PCs	% variance	training set	validation set^a^
Garda	7	99.97	10 samples (Garda)	73 samples
Sicilia	8	93.77	13 samples (Sicilia)	73 samples
Toscana	12	94.11	20 samples (Toscana)	73 samples
Lazio	7	99.82	11 samples (Lazio)	73 samples
Puglia	8	94.49	12 samples (Puglia)	73 samples
Umbria	5	97.23	7 samples (Umbria)	73 samples

a For the validation, all of the samples analyzed were employed.

Class boundaries were established for each predefined target class model on the basis of the values of Hotelling’s *T*^2^ and residual *Q* statistics. The classification criteria of the samples regarding each region were defined using a combination of the reduced *T*^2^ and *Q* statistic values. Thus, for a sample to be considered as belonging to a certain target class, both values must be less than 1.0.

Because the number of available samples from each Italian region was limited, each single-class model training was carried out employing all of the samples belonging to the concerned target class. Then, all 73 samples, both those belonging to the target class and those not, were used for validation purposes. All of the samples were correctly classified in five of the six single-class models, and the quality parameters such as sensitivity, specificity, precision, efficiency (accuracy), and area under the receiver operating characteristic curve (AUC) were equal to 1.00.^42^ The only model that misclassified one of the samples was the Garda model, in which a Garda sample was considered as non-Garda. Thus, in this model, the sensitivity, specificity, precision, efficiency (accuracy), and AUC were equal to 0.90, 1.00, 1.00, 0.99, and 0.95, respectively. The classification plots of each model are shown in the supplementary material (Supporting Information Figures S3–S8).

### Regional Signatures and GC × GC Identitation Potential

Based on the information shown in Table 3, it is possible to derive some conclusions about peculiar chemical traits specific to certain regions. For example, compounds #28 (*n*-hexane), #109 (1-penten-3-ol), and #386, #475, and #510 (all unidentified) are characteristic of the Garda region. In the same way, compound #141 ((*E*,*E*)-2,4-hexadienal) is specific of Sicilia samples, compound #95 (2-ethyl-2-hexenal) of Lazio, and compound #27 (*n*-octane) of Umbria. Further assignments could be identified as characteristic of more than one region, e.g., compound #245 (unidentified) is associated with Garda and Umbria. In the same way, following this assignment methodology, and considering the presence/absence of a few volatile compounds previously selected, a classification tree rule could be deduced to classify undoubtedly any sample of EVOO from any of the six considered regions. However, it might be beneficial to have a larger set of representative olive oil samples from each of the regions for such a classification tree to be sufficiently reliable.

The classification strategy proposed in this study is based on using the whole UT fingerprint of volatiles that is established by considering simultaneously all of the compounds that have been selected as characteristic of at least one of the regions concerned. In this way, the one-class SIMCA classification models are applied sequentially to any EVOO sample, regardless of geographic origin, so that the oil is assigned to one of the regions. This overall classification approach based on the use of UT fingerprinting, i.e., identitation, overcomes the main drawback for the routine application of single-step multivariate models.

Moreover, the strategy takes full advantage of the high-resolution power of GC × GC that effectively maps all detectable volatile components including (a) those related to major functional variables (e.g., olive cultivar,^13^ olive ripening stage,^16^ harvest year, and processing technology^12^), here playing a confounding role in regional classification; and (b) several potent odorants delineating EVOO sensory features. The latter might be masked by coelution phenomena occurring in 1D-GC^18^ while resulting in less effective identitation and poorly informative profiling processes.

## Supporting Information Available

List of analyzed samples grouped according to the production region, olive cultivars as declared in the label, certifications according to the EU quality schemes and/or conventional/organic production, and production year (Tables ST1–ST8); EVOO’s production areas/regions (Figures SF1–SF7) (PDF)
Compound name (XLSX)
