# Supplementary material for: Chromatographic Fingerprinting Enables Effective Discrimination and Identitation of High-Quality Italian Extra-Virgin Olive Oils
Source: J Agric Food Chem. 2021 Jul 28;69(31):8874–89. doi: 10.1021/acs.jafc.1c02981 (PMC8389832; doi:10.1021/acs.jafc.1c02981)

<u>Supporting information</u>

Chromatographic Fingerprinting Enables Effective Discrimination and *Identitation* of High-Quality Italian Extra-Virgin Olive Oils

Federico Stilo[1], Ana M. Jiménez-Carvelo[2,*], Erica Liberto[1], Carlo Bicchi[1], Stephen E. Reichenbach[3,4], Luis Cuadros-Rodríguez[2], and Chiara Cordero[1,*]

[1] Dipartimento di Scienza e Tecnologia del Farmaco, Università degli Studi di Torino, Turin, Italy

[2] Department of Analytical Chemistry, Faculty of Science, University of Granada, Av. Fuentenueva S/N, E-18071, Granada, Spain

[3] University of Nebraska-Lincoln, NE USA

[4] GC Image LLC, Lincoln, NE USA

* Corresponding authors:

Prof. Chiara Cordero – Dipartimento di Scienza e Tecnologia del Farmaco, Università di Torino; Via Pietro Giuria 9, I-10125 Torino, Italy

e-mail: chiara.cordero@unito.it; phone: +39 011 6707172

Dr. Ana M. Jiménez Carvelo - Department of Analytical Chemistry, Faculty of Sciences, University of Granada, Av. Fuentenueva S/N, E-18071, Granada, Spain

e-mail: amariajc@ugr.es; phone: +34 958240797

Supplementary Table 1 –ST1*:* List of analyzed samples grouped according to production Region, olive cultivars as declared in the label, certifications according to EU quality schemes and/or conventional/organic production, and production year. All samples were collected within the Project Violin – Progetto AGER [19] and certified as EVOO by accredited laboratories and official sensory panel.

**Origin**	**ID#**	**Olive Cultivars**	**Certifications**	**Production**
Garda	G1	*Casaliva, Leccino*	PDO	2017
	G2	*Casaliva, Leccino*	PDO	2017
	G3	*Casaliva, Leccino*	PDO	2017
	G4	*Casaliva, Leccino, Moraiolo, Pendolino, Frantoio*	PDO	2017
	G5	*Casaliva*	PDO	2017
	G6	*Casaliva (>70%), Frantoio, Leccino*	Organic	2017
	G7	*Coratina*		2017
	G8	*Grignano*		2018
	G9	*Grignano, Favarol, Pendolino, Trepp*	PDO	2017
	G10	*Blend*	Organic	2017
Sicilia	S1	*Nocellara del Belice*		2016
	S2	*Cerasuola, Nocellara del Belice, Biancolilla*	PGI	2017
	S3	*Nocellara del Belice*	PDO	2017
	S4	*Biancolilla*	PDO	2017
	S5	*Nocellara del Belice*	PDO	2017
	S6	*Tonda Iblea*	PDO	2017
	S7	*Nocellara Messinese*	Organic	2017
	S8	*Nocellara Etnea*	Organic	2017
	S9	*Nocellara Etnea*		2018
	S10	*Nocellara del Belice*		2017
	S11	*Nocellara del Belice*		2018
	S12	*Biancolilla*		2018
	S13	*Cerasuola*		2017
Toscana	T1	*Leccino, Moraiolo, Frantoio, Maurino*	PGI	2017
	T2	*Moraiolo, Frantoio, Leccino*	PGI	2017
	T3	*Frantoio, Leccino*	PGI	2017
	T4	*Moraiolo, Leccino, Frantoio, Correggiolo*	PGI	2017
	T5	*Corregiolo (50%), Leccio del Corno, Pendolino, Maurino*	PGI	2017
	T6	*Moraiolo, Leccino, Frantoio*	PGI	2017
	T7	*Frantoio*	PGI	2017
	T8	*Olivastra Saggianese*	PDO	2017
	T9	*Frantoio, Leccino, Moraiolo, Pendolino, Morchiaio, Leccio del Corno*	PDO	2017
	T10	*Frantoio, Moraiolo, Leccino, Pendolino*	PDO	2017
	T11	*Correggiolo (95%), Leccino, Frantoiano*	PDO	2017

	T12	*Moraiolo*		2017
	T13	*Frantoio*		2017
	T14	*Frantoio*		2017
	T15	*Leccio del corno*		2017
	T16	*Leccio del corno*		2017
	T17	*Moraiolo*	Organic	2017
	T18	*Pendolno*	Organic	2017
	T19	*Frantoio (50%), Leccino, Moraiolo, Pendolino*		2017
	T20	*Blend*	Organic	2017
Lazio	L1	*Blend*	Organic	2016
	L2	*Leccino*		2016
	L3	*Blend*		2016
	L4	*Caninese*	PDO	2017
	L5	*Caninese*	PDO	2017
	L6	*Caninese, Frantoio, Maurino*	Organic	2017
	L7	*Blend*	Organic	2017
	L8	*Blend*		2017
	L9	*Caninese, Frantoio, Leccino*		2017
	L10	*Itrana*	Organic	2017
	L11	*Itrana*		2017

Supplementary Table 1 – ST1 continued

Umbria	U1	*Blend*		2016
	U2	*Leccino (60%), Frantoio, Moraiolo*	PDO	2017
	U3	*Moraiolo, Frantoio, Leccino*	PDO	2017
	U4	*Moraiolo, Frantoio, Leccino*		2017
	U5	*Moraiolo, Frantoio, Leccino*		2017
	U6	*Moraiolo, Frantoio, Leccino*		2017
	U7	*Leccino, Frantoio, Moraiolo*		2017
Puglia	P1	*Coratina, Regina della Puglia*	PDO	2017
	P2	*Coratina*	PDO	2017
	P3	*Ogliarola garganica*	Organic	2017
	P4	*Peranzana*	Organic	2017
	P5	*Coratina*	Organic	2017
	P6	*Coratina*	Organic	2017
	P7	*Coratina*		2017
	P8	*Frantoio*		2017
	P9	*Coratina*		2017
	P10	*Coratina*		2017
	P11	*Peranzana*		2017
	P12	*Ogliarola*		2017

Supplementary Table 2 – ST2. List of untargeted and targeted peak regions included in the UT template, together with their linear retention index (I^T) and retention times in the two analytical dimensions (^1t_R, ^2t_R).

Provided as Excel file.

Supplementary Table 3 - ST3. Selected variables/volatile compounds from Garda.

No. Variable	Compound	No. Variable	Compound
5	α-Pinene	258	(unidentified)
8	α-Copaene	262	(unidentified)
12	Terpinene	267	(unidentified)
20	Pentanal	274	(unidentified)
26	Nonanal	275	(unidentified)
28	n-Hexane	276	(unidentified)
34	Limonene	278	(unidentified)
39	Hexanal	285	(unidentified)
47	Ethyl acetate	294	(unidentified)
54	Cyclohexane	299	(unidentified)
60	Butanoic butanoate	305	(unidentified)
73	4-Hydroxy-2-hexenoic acid lactone	314	(unidentified)
85	3,4-Diethyl-1,5-hexadiene (meso)	318	(unidentified)
108	1-Penten-3-one	323	(unidentified)
109	1-Penten-3-ol	331	(unidentified)
115	1-Methoxyhexane	340	(unidentified)
116	1-Hexanol	374	(unidentified)
127	1,4-Pentadiene	377	(unidentified)
132	(Z)-3-Hexenyl acetate	382	(unidentified)
133	(Z)-3-Hexen-1-ol	386	(unidentified)
136	(Z)-2-Hexenal	388	(unidentified)
138	(E,Z)-3,7-Decadiene	400	(unidentified)
140	(E,E)-3,7-Decadiene	413	(unidentified)
141	(E,E)-2,4-Hexadienal	418	(unidentified)
144	(E)-ß-Ocimene	422	(unidentified)
148	(E)-2-Penten-1-ol	430	(unidentified)
151	(E)-2-Hexen-1-ol	454	(unidentified)
155	(5Z)-3-Ethyl-1,5-octadiene	458	(unidentified)
156	(5E)-3-Ethyl-1,5-octadiene	475	(unidentified)
187	(unidentified)	504	(unidentified)
188	(unidentified)	505	(unidentified)
213	(unidentified)	510	(unidentified)

215	(unidentified)	523	(unidentified)
227	(unidentified)	549	(unidentified)
238	(unidentified)	554	(unidentified)
243	(unidentified)	555	(unidentified)
245	(unidentified)	573	(unidentified)
254	(unidentified)	588	(unidentified)

Supplementary Table 4 – ST4. Selected variables/volatile compounds from Sicilia.

No. Variable	Compound	No. Variable	Compound
8	α-Copaene	258	(unidentified)
20	Pentanal	267	(unidentified)
39	Hexanal	275	(unidentified)
47	Ethyl acetate	285	(unidentified)
54	Cyclohexane	299	(unidentified)
56	Butyl isobutyrate	305	(unidentified)
73	4-Hydroxy-2-hexenoic acid lactone	318	(unidentified)
84	3,4-Diethyl-1,5-hexadiene (RS+SR)	331	(unidentified)
116	1-Hexanol	340	(unidentified)
132	(Z)-3-Hexenyl acetate	349	(unidentified)
133	(Z)-3-Hexen-1-ol	374	(unidentified)
136	(Z)-2-Hexenal	377	(unidentified)
138	(E,Z)-3,7-Decadiene	382	(unidentified)
140	(E,E)-3,7-Decadiene	413	(unidentified)
141	(E,E)-2,4-Hexadienal	418	(unidentified)
147	(E)-2-Pentenal	422	(unidentified)
148	(E)-2-Penten-1-ol	424	(unidentified)
151	(E)-2-Hexen-1-ol	454	(unidentified)
155	(5Z)-3-Ethyl-1,5-octadiene	458	(unidentified)

156	(5E)-3-Ethyl-1,5-octadiene	498	(unidentified)
175	(unidentified)	505	(unidentified)
188	(unidentified)	523	(unidentified)
190	(unidentified)	550	(unidentified)
201	(unidentified)	554	(unidentified)
205	(unidentified)	555	(unidentified)
213	(unidentified)	561	(unidentified)
227	(unidentified)	573	(unidentified)
238	(unidentified)	588	(unidentified)
243	(unidentified)		
254	(unidentified)		

Supplementary Table 5 – ST5. Selected variables/volatile compounds from Toscana.

No. Variable	Compound	No. Variable	Compound
5	α-Pinene	254	(unidentified)
8	α-Copaene	262	(unidentified)
20	Pentanal	267	(unidentified)
26	Nonanal	274	(unidentified)
34	Limonene	275	(unidentified)
37	Hexyl acetate	276	(unidentified)
39	Hexanal	285	(unidentified)
47	Ethyl acetate	299	(unidentified)
54	Cyclohexane	305	(unidentified)
60	Butanoic butanoate	314	(unidentified)
73	4-Hydroxy-2-hexenoic acid lactone	318	(unidentified)
95	2-Ethyl-2-hexenal	323	(unidentified)
96	2-Ethyl-1-hexanol	331	(unidentified)
108	1-Penten-3-one	340	(unidentified)
115	1-Methoxyhexane	349	(unidentified)
116	1-Hexanol	374	(unidentified)
132	(Z)-3-Hexenyl acetate	377	(unidentified)
133	(Z)-3-Hexen-1-ol	382	(unidentified)
136	(Z)-2-Hexenal	386	(unidentified)
138	(E,Z)-3,7-Decadiene	388	(unidentified)
140	(E,E)-3,7-Decadiene	396	(unidentified)
147	(E)-2-Pentenal	413	(unidentified)
148	(E)-2-Penten-1-ol	418	(unidentified)
151	(E)-2-Hexen-1-ol	422	(unidentified)
152	(E)-2-Heptenal	454	(unidentified)
155	(5Z)-3-Ethyl-1,5-octadiene	458	(unidentified)
156	(5E)-3-Ethyl-1,5-octadiene	504	(unidentified)
175	(unidentified)	505	(unidentified)
188	(unidentified)	523	(unidentified)
201	(unidentified)	529	(unidentified)
213	(unidentified)	538	(unidentified)
215	(unidentified)	554	(unidentified)

227	(unidentified)	555	(unidentified)
232	(unidentified)	573	(unidentified)
238	(unidentified)	588	(unidentified)
243	(unidentified)		

Supplementary Table 6 – ST6. Selected variables/volatile compounds from Lazio.

No. Variable	Compound	No. Variable	Compound
5	α-Pinene	213	(unidentified)
8	α-Copaene	215	(unidentified)
20	Pentanal	232	(unidentified)
26	Nonanal	238	(unidentified)
27	n-Octane	244	(unidentified)
28	n-Hexane	254	(unidentified)
34	Limonene	268	(unidentified)
37	Hexyl acetate	274	(unidentified)
39	Hexanal	276	(unidentified)
46	Ethyl benzoate	285	(unidentified)
47	Ethyl acetate	299	(unidentified)
53	Cyclopentane	305	(unidentified)
54	Cyclohexane	314	(unidentified)
56	Butyl isobutyrate	318	(unidentified)
60	Butanoic butanoate	323	(unidentified)
66	Acetonitrile	331	(unidentified)
70	6-Methyl-5-hepten-2-one	340	(unidentified)
73	4-Hydroxy-2-hexenoic acid lactone	349	(unidentified)
84	3,4-Diethyl-1,5-hexadiene (RS+SR)	374	(unidentified)
85	3,4-Diethyl-1,5-hexadiene (meso)	375	(unidentified)
95	2-Ethyl-2-hexenal	377	(unidentified)
108	1-Penten-3-one	382	(unidentified)
109	1-Penten-3-ol	388	(unidentified)
116	1-Hexanol	400	(unidentified)
126	1-Butanol	413	(unidentified)
127	1,4-Pentadiene	418	(unidentified)
132	(Z)-3-Hexenyl acetate	422	(unidentified)
133	(Z)-3-Hexen-1-ol	445	(unidentified)
138	(E,Z)-3,7-Decadiene	454	(unidentified)
139	(E,Z)-2,4-Hexadienal	457	(unidentified)
140	(E,E)-3,7-Decadiene	458	(unidentified)
144	(E)-ß-Ocimene	480	(unidentified)

148	(E)-2-Penten-1-ol	498	(unidentified)
151	(E)-2-Hexen-1-ol	504	(unidentified)
155	(5Z)-3-Ethyl-1,5-octadiene	505	(unidentified)
156	(5E)-3-Ethyl-1,5-octadiene	510	(unidentified)
175	(unidentified)	523	(unidentified)
178	(unidentified)	529	(unidentified)
201	(unidentified)	555	(unidentified)
205	(unidentified)	573	(unidentified)
210	(unidentified)	588	(unidentified)

Supplementary Table 7 – ST7. Selected variables/volatile compounds from Puglia.

No. Variable	Compound	No. Variable	Compound
5	α-Pinene	254	(unidentified)
8	α-Copaene	262	(unidentified)
20	Pentanal	267	(unidentified)
26	Nonanal	274	(unidentified)
32	Methyl benzoate	275	(unidentified)
34	Limonene	278	(unidentified)
39	Hexanal	285	(unidentified)
46	Ethyl benzoate	299	(unidentified)
47	Dodecanal	305	(unidentified)
50	Diethyl phtalate	314	(unidentified)
54	Cyclohexane	318	(unidentified)
60	Butanoic butanoate	323	(unidentified)
65	Benzaldehyde	331	(unidentified)
66	Acetonitrile	340	(unidentified)
73	4-Hydroxy-2-hexenoic acid lactone	341	(unidentified)
84	3,4-Diethyl-1,5-hexadiene (RS+SR)	349	(unidentified)
96	2-Ethyl-1-hexanol	359	(unidentified)
108	1-Penten-3-one	374	(unidentified)
116	1-Hexanol	377	(unidentified)
132	(Z)-3-Hexenyl acetate	382	(unidentified)
133	(Z)-3-Hexen-1-ol	396	(unidentified)
134	(Z)-2-Pentenal	400	(unidentified)
136	(Z)-2-Hexenal	413	(unidentified)
138	(E,Z)-3,7-Decadiene	418	(unidentified)
140	(E,E)-3,7-Decadiene	422	(unidentified)
148	(E)-2-Penten-1-ol	454	(unidentified)
151	(E)-2-Hexen-1-ol	458	(unidentified)
155	(5Z)-3-Ethyl-1,5-octadiene	468	(unidentified)
156	(5E)-3-Ethyl-1,5-octadiene	480	(unidentified)
160	(unidentified)	498	(unidentified)
188	(unidentified)	504	(unidentified)
205	(unidentified)	505	(unidentified)

210	(unidentified)	555	(unidentified)
213	(unidentified)	573	(unidentified)
232	(unidentified)	588	(unidentified)
238	(unidentified)		

Supplementary Table 8 – ST8. Selected variables/volatile compounds from Umbria.

No. Variable	Compound	No. Variable	Compound
5	α-Pinene	245	(unidentified)
8	α-Copaene	258	(unidentified)
20	Pentanal	267	(unidentified)
27	n-Octane	268	(unidentified)
34	Limonene	274	(unidentified)
37	Hexyl acetate	276	(unidentified)
39	Hexanal	280	(unidentified)
46	Ethyl benzoate	285	(unidentified)
53	Cyclopentane	299	(unidentified)
54	Cyclohexane	305	(unidentified)
56	Butyl isobutyrate	314	(unidentified)
60	Butanoic butanoate	318	(unidentified)
66	Acetonitrile	323	(unidentified)
70	6-Methyl-5-hepten-2-one	331	(unidentified)
73	4-Hydroxy-2-hexenoic acid lactone	340	(unidentified)
84	3,4-Diethyl-1,5-hexadiene (RS+SR)	349	(unidentified)
108	1-Penten-3-one	374	(unidentified)
116	1-Hexanol	375	(unidentified)
132	(Z)-3-Hexenyl acetate	377	(unidentified)
133	(Z)-3-Hexen-1-ol	382	(unidentified)
138	(E,Z)-3,7-Decadiene	388	(unidentified)
139	(E,Z)-2,4-Hexadienal	413	(unidentified)
140	(E,E)-3,7-Decadiene	418	(unidentified)
148	(E)-2-Penten-1-ol	422	(unidentified)
151	(E)-2-Hexen-1-ol	454	(unidentified)
155	(5Z)-3-Ethyl-1,5-octadiene	457	(unidentified)
156	(5E)-3-Ethyl-1,5-octadiene	458	(unidentified)
166	(unidentified)	498	(unidentified)
175	(unidentified)	504	(unidentified)
201	(unidentified)	505	(unidentified)
205	(unidentified)	523	(unidentified)
213	(unidentified)	529	(unidentified)

232	(unidentified)	555	(unidentified)
238	(unidentified)	573	(unidentified)
244	(unidentified)	588	(unidentified)

Supplementary Figure 1 – SF1. EVOO's production areas/Regions: purple "S" samples are from Sicily - Sicilia, yellow "P" samples are from Puglia, orange "L" samples are from Lazio, light blue "U" samples are from Umbria, green "T" samples are from Tuscany – Toscana, and pink "G" samples are from Garda lake area.

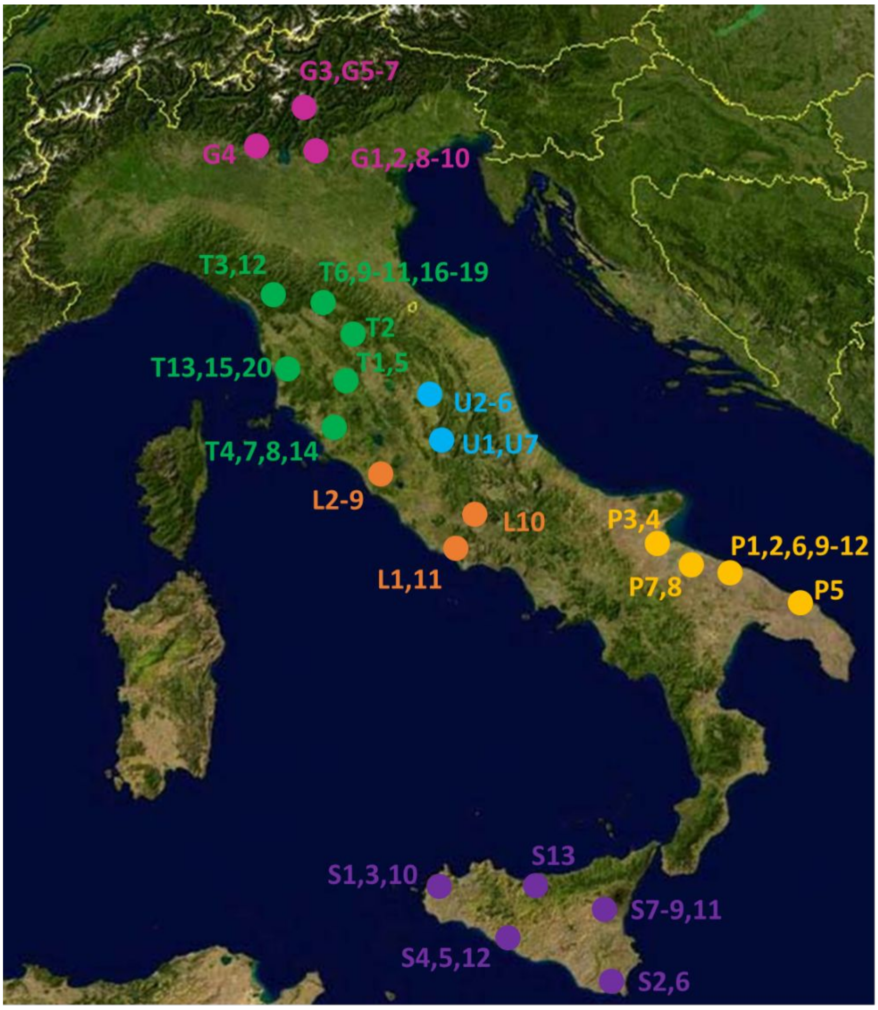

Supplementary Figure 2 –SF2. SIMCA model from Garda (validation step)

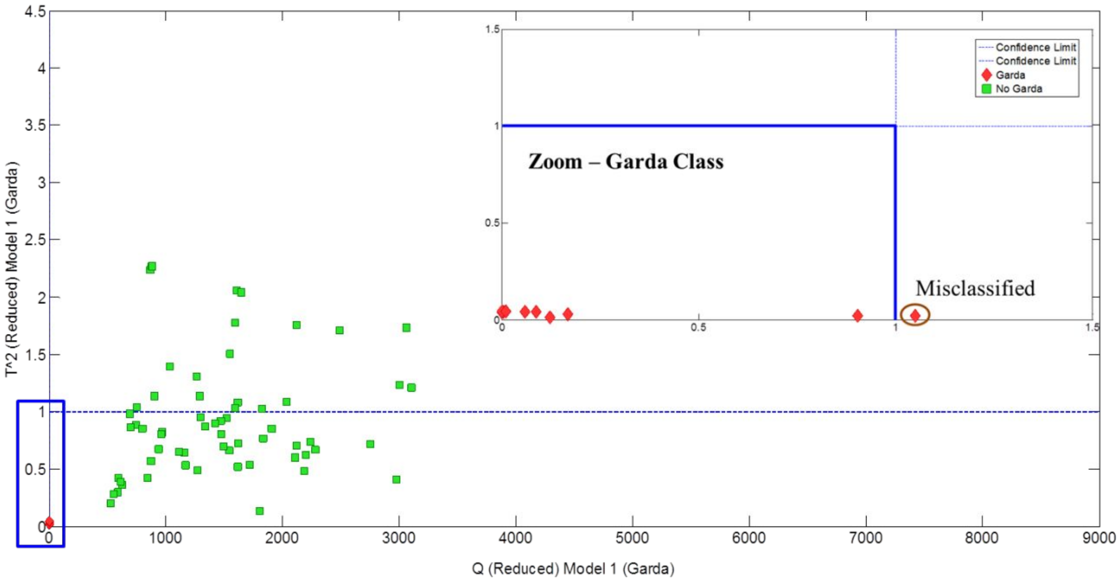

Supplementary Figure 3 –SF3. SIMCA model from Sicilia (validation step)

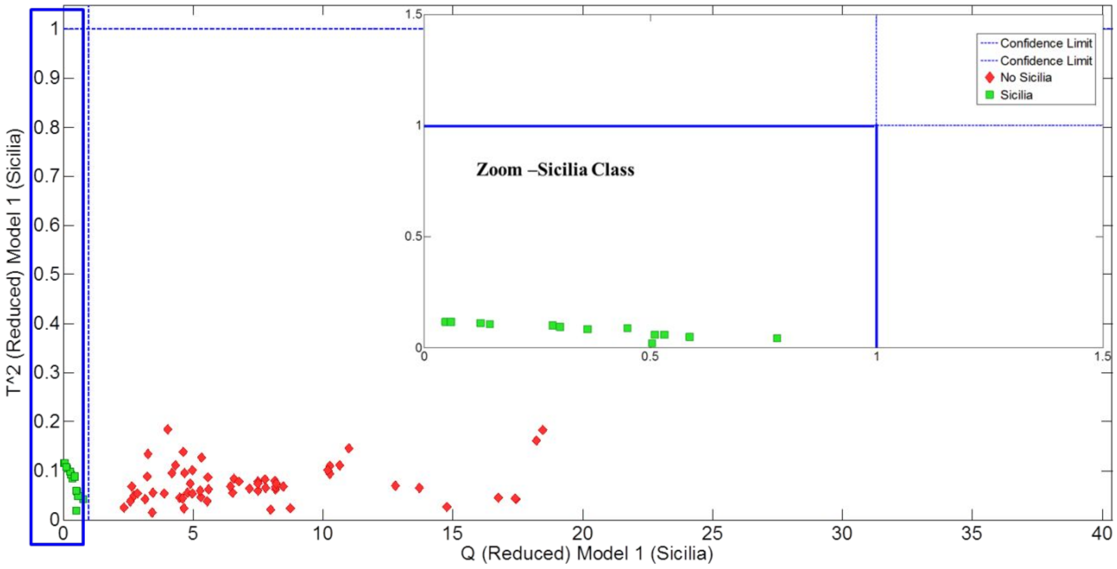

Supplementary Figure 4 –SF4. SIMCA model from Toscana (validation step)

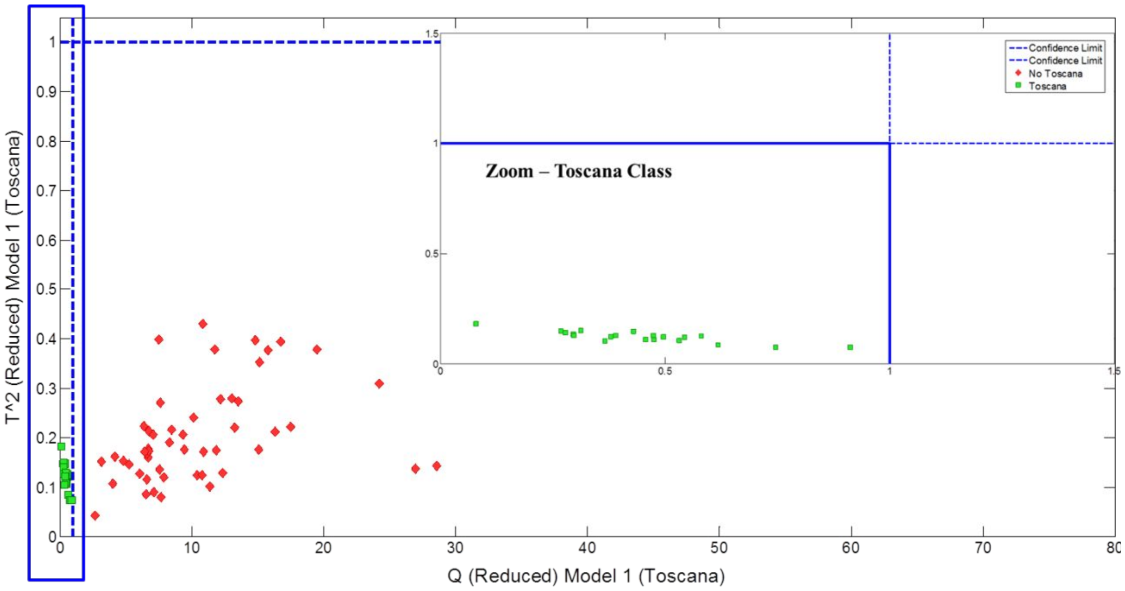

Supplementary Figure 5 –SF5. SIMCA model from Lazio (validation step)

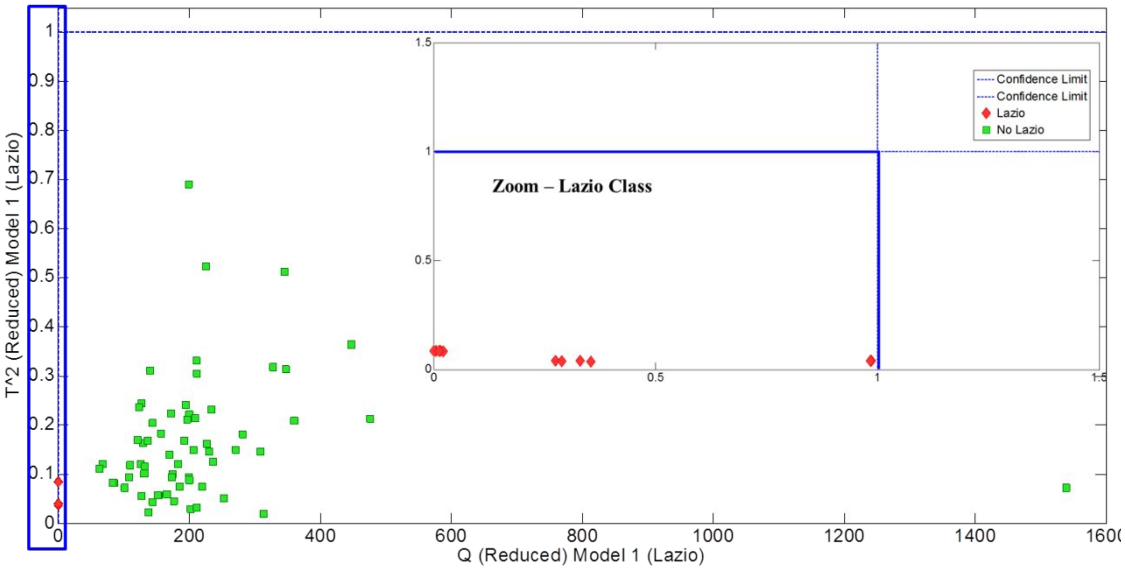

Supplementary Figure 6 –SF6. SIMCA model from Puglia (validation step)

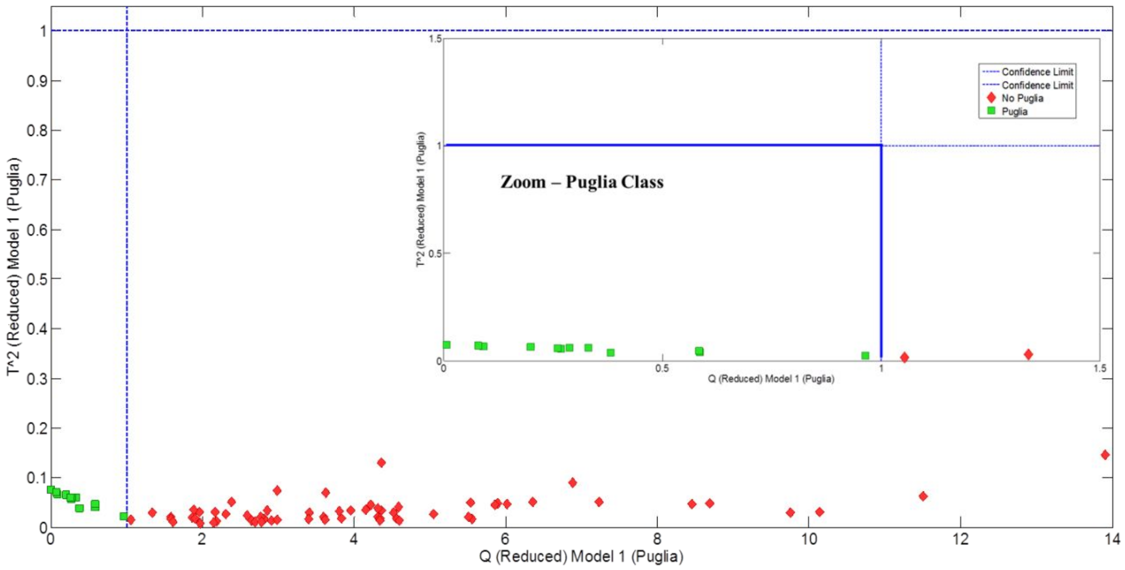

Supplementary Figure 7–SF7. SIMCA model from Umbria (validation step)

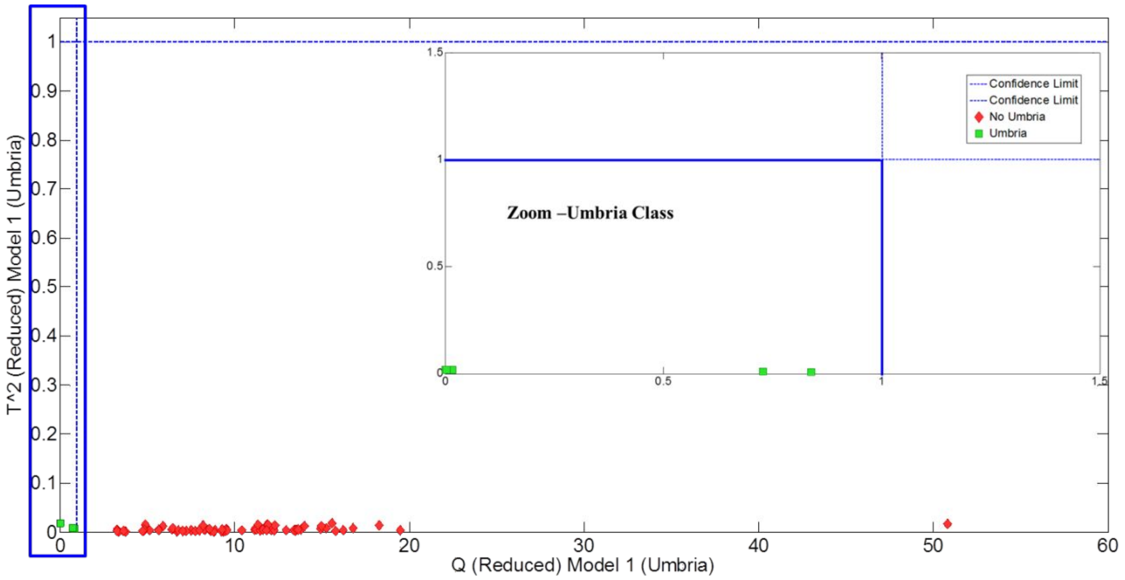

Supplement: Supplementary file 1 — jf1c02981_si_001.pdf [file jf1c02981_si_001.pdf]
